# Alternative dimethylsulfoniopropionate biosynthesis enzymes in diverse and abundant microorganisms

**DOI:** 10.1038/s41564-024-01715-9

**Published:** 2024-06-11

**Authors:** Jinyan Wang, Andrew R. J. Curson, Shun Zhou, Ornella Carrión, Ji Liu, Ana R. Vieira, Keanu S. Walsham, Serena Monaco, Chun-Yang Li, Qing-Yu Dong, Yu Wang, Peter Paolo L. Rivera, Xiao-Di Wang, Min Zhang, Libby Hanwell, Matthew Wallace, Xiao-Yu Zhu, Pedro N. Leão, David J. Lea-Smith, Yu-Zhong Zhang, Xiao-Hua Zhang, Jonathan D. Todd

**Affiliations:** 1 Frontiers Science Center for Deep Ocean Multispheres and Earth System, and College of Marine Life Sciences, Ocean University of China, Qingdao, China; 2 School of Biological Sciences, University of East Anglia, Norwich Research Park, Norwich, UK; 3 Key Laboratory of Marine Chemistry Theory and Technology, Ministry of Education, Ocean University of China, Qingdao, China; 4 School of Marine Science and Engineering, Qingdao Agricultural University, Qingdao, China; 5 Interdisciplinary Centre of Marine and Environmental Research (CIIMAR/CIMAR), University of Porto, Matosinhos, Portugal; 6 School of Pharmacy, University of East Anglia, Norwich Research Park, Norwich, UK; 7 State Key Lab of Microbial Technology, Marine Biotechnology Research Center, Shandong University, Qingdao, China; 8 Laboratory for Marine Ecology and Environmental Science, Laoshan Laboratory, Qingdao, China

**Keywords:** Bacteria, Marine microbiology

## Abstract

Dimethylsulfoniopropionate (DMSP) is an abundant marine organosulfur compound with roles in stress protection, chemotaxis, nutrient and sulfur cycling and climate regulation. Here we report the discovery of a bifunctional DMSP biosynthesis enzyme, DsyGD, in the transamination pathway of the rhizobacterium *Gynuella sunshinyii* and some filamentous cyanobacteria not previously known to produce DMSP. DsyGD produces DMSP through its N-terminal DsyG methylthiohydroxybutyrate *S*-methyltransferase and C-terminal DsyD dimethylsulfoniohydroxybutyrate decarboxylase domains. Phylogenetically distinct DsyG-like proteins, termed DSYE, with methylthiohydroxybutyrate *S*-methyltransferase activity were found in diverse and environmentally abundant algae, comprising a mix of low, high and previously unknown DMSP producers. Algae containing *DSYE*, particularly bloom-forming *Pelagophyceae* species, were globally more abundant DMSP producers than those with previously described DMSP synthesis genes. This work greatly increases the number and diversity of predicted DMSP-producing organisms and highlights the importance of *Pelagophyceae* and other *DSYE*-containing algae in global DMSP production and sulfur cycling.

## Abstract (web summary)

The number and diversity of organisms known to produce the environmentally important sulfur compound dimethylsulfoniopropionate (DMSP) is expanded with the discovery of a family of DMSP synthesis enzymes.

## Main

Petagrams of dimethylsulfoniopropionate (DMSP) are made annually in Earth’s surface waters, with potentially much more in marine aphotic, sediment and coastal settings^1–5^. DMSP is an anti-stress compound^6–9^ produced to millimolar concentrations within diverse algae, corals, bacteria and some angiosperms^10^. When released into the environment, DMSP is also a major source of carbon and sulfur to marine microorganisms^11^ and of climate-cooling gases and/or signalling molecules^11^, including dimethyl sulfide (DMS) and methanethiol (MeSH), via DMSP catabolism^12^.

**Fig. 1:**
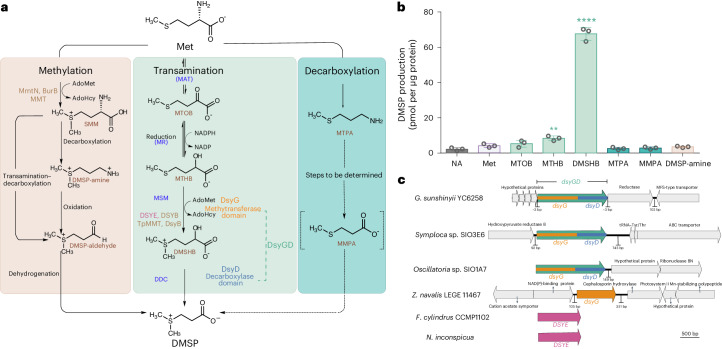
DMSP biosynthesis genes, enzymes and pathways. **a**, The ‘methylation’ pathway in some higher plants with the methionine (Met) *S*-methyltransferase (MMT) and bacteria containing MmtN or another methyltransferase (BurB) (left); the ‘transamination’ pathway in algae, bacteria and corals with DSYB/DsyB, DsyGD/DsyG, DSYE and/or TpMMT (middle); and the ‘decarboxylation’ pathway in *Crypthecodinium cohnii* (right). The pathways are named after their first reaction step (in larger font). AdoMet, *S*-adenosylmethionine; AdoHcy, *S*-adenosylhomocysteine; NADP, nicotine adenine dinucleotide phosphate; MAT, methionine aminotransferase; MR, MTOB reductase; MSM, MTHB *S*-methyltransferase; DDC, DMSHB decarboxylase; SMM, *S*-methylmethionine; MTOB, 4-methylthio-2-oxybutyrate; MTHB, 4-methylthio-2-hydroxybutyrate; DMSHB, 4-dimethylsulfonio-2-hydroxybutyrate; MTPA, 3-methylthiopropylamine; MMPA, 3-methylmercaptopropionate. The enzymes and domains identified here are coloured to match their corresponding genes in **c**. **b**, DMSP accumulation in *G.* *sunshinyii* incubated with DMSP synthesis intermediates (0.5 mM) or nothing added (NA, control). The results show the mean values of three independent biological replicates with error bars indicating standard deviations. The statistically significant differences compared with control conditions were determined using a two-sided Student’s *t*-test (**P = 0.0025 and ****P = 7.74 × 10^−6^). **c**, The genomic location of *dsyGD*/*dsyG* in DMSP-producing bacteria. The algal *DSYE* transcripts are included for size comparison. For *Oscillatoria* sp. SIO1A7, *dsyGD* is located at the start of the contig. MFS, major facilitator superfamily; tRNA, transfer RNA; ribonuclease BN, ribonuclease from *Escherichia coli* strain BN; ABC, ATP-binding cassette. Source data

Recent work has categorized DMSP producers into low (<50 mM) and high (≥50 mM) accumulators^13^ and identified key genes encoding single-domain *S*-methyltransferase enzymes involved in, and that are robust indicators for, DMSP synthesis in diverse algae (*DSYB* and *TpMMT*) and bacteria (*dsyB*, *mmtN* and *burB*) (Fig. 1a)^1,14–18^. However, many known DMSP-producing algae^19^, bacteria^14^ and plants^20–23^ lack these DMSP synthesis genes and probably contain alternative DMSP synthesis enzymes. Thus, despite some recent attempts^9,24–26^, it is currently challenging to predict from omics data which organisms are important DMSP producers in environmental samples. In this Article, we elucidate and characterize the activity, biodiversity, potential role and environmental importance of previously unknown DMSP synthesis enzymes in cyanobacteria, other bacteria and eukaryotic algae and identify additional and important global DMSP producers.

## Results

### *G.sunshinyii* makes DMSP by the transamination pathway

This study initially focused on *G.* *sunshinyii*, a rhizobacterium with anti-fungal activity isolated from the salt marsh plants *Carex scabrifolia* and *Spartina alterniflora*^27,28^. The *S.* *alterniflora* rhizosphere is rich in DMSP produced by this cordgrass^29–32^ and microbial DMSP cycling^21,33–35^. DMSP was also found in *C.* *scabrifolia* leaves, roots and rhizosphere samples (ranging from 5.51 ± 0.15 to 6.92 ± 0.13 nmol DMSP g^−1^; Supplementary Fig. 1). It was possible that these plants fed DMSP to *G.* *sunshinyii* in return for favourable bacterial traits and metabolites, for example, activity against fungal pathogens^28,36–38^. However, *G.* *sunshinyii* (strain YC6258 (ref. ^27^)) could not use DMSP as a sole carbon source nor liberate DMS or MeSH from DMSP, consistent with its genome lacking all known DMSP lyase genes^39–47^ and the DMSP demethylation gene *dmdA*^48^. Instead, *G.* *sunshinyii* produced DMSP when grown without added organosulfur compounds and at levels approximately threefold higher than the model DMSP-producing bacterium *Labrenzia aggregata*^1^ (101.11 ± 6.64 and 35.38 ± 3.94 pmol µg^−1^ of protein, respectively). DMSP synthesis in *G.* *sunshinyii* was investigated because its genome lacked all known DMSP synthesis genes.

Incubation of *G.* *sunshinyii* cells with DMSP synthesis intermediates from the transamination pathway^49,50^ (Fig. 1a), 4-methylthio-2-hydroxybutyrate (MTHB) and 4-dimethylsulfonio-2-hydroxybutyrate (DMSHB), significantly enhanced DMSP accumulation by 2- and 30-fold, respectively, whereas those from the methylation and decarboxylation pathways had no significant effects compared to controls with no added intermediates (Fig. 1b). DMSHB probably resulted in higher DMSP levels because it is specific to the transamination pathway for DMSP synthesis^49^, whereas MTHB is a substrate in competing pathways, for example, in methionine (Met) salvage^51^. Furthermore, *G. sunshinyii* cell extracts displayed in vitro MTHB *S*-methyltransferase (MSM) and DMSHB decarboxylase (DDC) activities (39.11 ± 0.21 and 9.23 ± 0.19 pmol DMSP per µg of protein per hour, respectively). These data implied that *G.* *sunshinyii* synthesized DMSP via the transamination pathway.

### Identification of a bifunctional DMSP synthesis enzyme

**Fig. 2:**
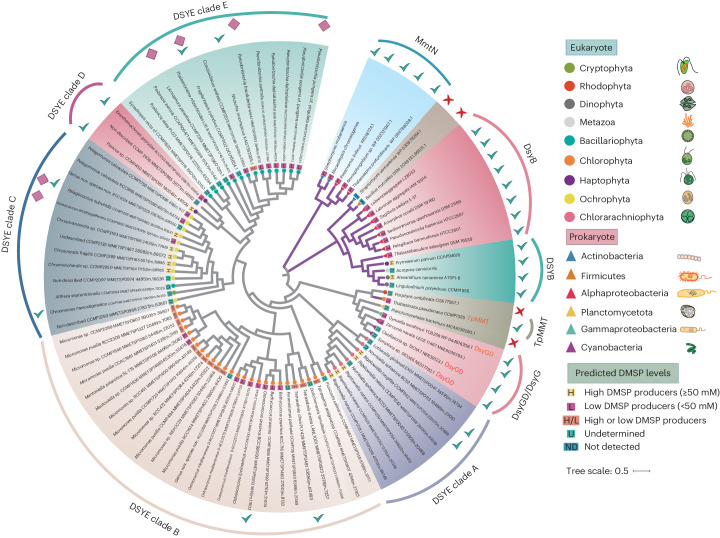
Maximum-likelihood phylogenetic tree of DsyG/DsyGD and DSYE proteins and related methyltransferases. The tree was constructed in MEGA X (ref. ^87^) using the sequences of previously characterized *S*-methyltransferases involved in DMSP synthesis (Supplementary Table 10) and others shown to be not functional^1,14–16^, including those from this study and homologues from MMETSP. Where proteins were multi-domain (labelled DsyGD), only the DsyG *S*-methyltransferase domain was analysed. Experimentally ratified (as functional) MSM or MmtN are marked with green ticks, while non-functional *S*-methyltransferases are labelled with a red cross. Eukaryotic (circles) and prokaryotic (triangles) proteins are coloured according to the taxonomy described in the key. The organisms containing both DSYE and DSYB are indicated with a rhombus. The proteins identified and discussed from previous studies are marked with purple branches. The predicted intracellular DMSP levels of the organisms^13^ are also indicated.

A *G.* *sunshinyii* genomic library was constructed and screened for MSM activity in *Rhizobium leguminosarum*. One from 3,000 clones screened (termed pJDT0020) conferred MSM activity. Unlike *dsyB*/*DSYB* clones^1,15^, pJDT0020 conferred MSM activity in *Escherichia coli* (2.51 ± 0.12 pmol DMSP per µg of protein per hour), but intriguingly, also DDC activity (0.74 ± 0.08 pmol DMSP per µg of protein per hour), implying that *G.* *sunshinyii* contained a DMSP synthesis gene cluster. The ~30 kb insert in pJDT0020 contained a gene, termed *dsyGD*, adjacent to another predicted to encode a 4-methylthio-2-oxobutyrate (MTOB) reductase (Fig. 1a,c). DsyGD is a 494 amino acid protein with two domains (Supplementary Fig. 2). The N-terminal methyltransferase domain (Pfam PF08241.15, 76–175 amino acids), termed DsyG, had 31% amino acid identity to *Thalassiosira pseudonana* TpMMT^16^, was phylogenetically distinct and formed a separate clade from this and all other known *S*-methyltransferases involved in DMSP synthesis^10^ (Fig. 2). The DsyGD C-terminal domain (Pfam PF04115.15, 320–469 amino acids), termed DsyD, was similar to an ureidoglycolate lyase domain and was predicted to be a DMSHB decarboxylase (Supplementary Fig. 2).

**Fig. 3:**
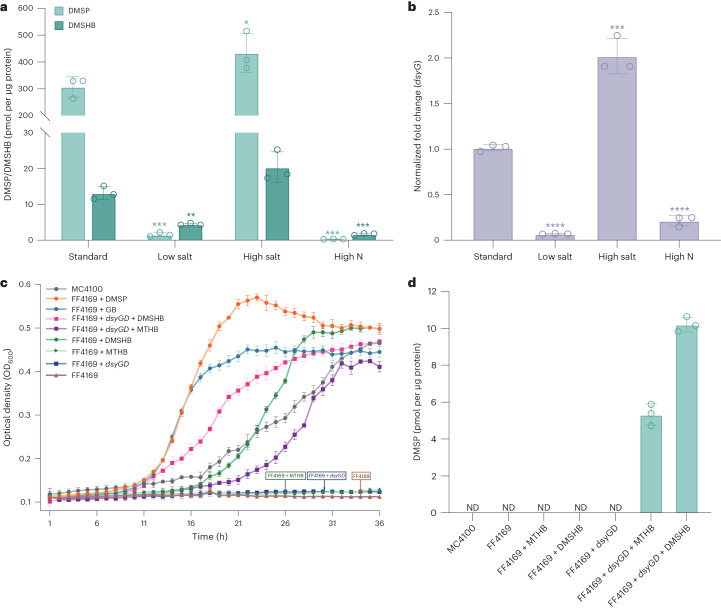
Regulation of *G. sunshinyii* DMSP synthesis and role of DsyGD in salt tolerance. **a**, *G.* *sunshinyii* DMSP and DMSHB accumulation measured by GC. **b**, *dysG* transcription from cultures grown under standard conditions (35 PSU MBM and 0.5 mM NH_4_Cl), low salt (5 PSU), high salt (50 PSU) or high nitrogen (10 mM NH_4_Cl). DMSP and DMSHB values in **a** represent the mean of three independent biological replicates with the error bars indicating the respective standard deviations. For **a**, statistically significant differences compared with control conditions were determined using a two-sided Student’s *t*-test (DMSP group, low salt: ***P = 0.0002; high salt: *P = 0.0409; high N: *** *P* = 0.0002. DMSHB group, low salt: **P = 0.0013 and ***P = 0.0004). For the RT–qPCR assays in **b**, the mean values of three technical replicates for each of three independent biological replicates are shown. The error bars indicate standard deviation. For **b**, statistically significant differences compared with control conditions were determined using a two-sided Student’s *t*-test (low salt: ****P = 1.31 × 10^−6^; high salt: *** *P* = 0.0009; high N: ****P = 3.219 × 10^−5^). **c**, Growth of wild type *E.* *coli* MC4100, the salt-sensitive *E.* *coli otsA*^*−*^mutant strain FF4169 (deficient in trehalose production) and FF4169 strains expressing cloned *dsyGD* was monitored in media containing 0.5 M NaCl alone or with 1 mM GB, DMSP or DMSP synthesis intermediates (MTHB and DMSHB). The arrows indicate the three strains that did not grow. The values shown represent the mean of three biological replicates with the error bars indicating the respective standard deviations. **d**, DMSP levels in selected cells after the 36 h incubation experiments shown in **c**. The mean values of three biological replicates are shown with the error bars indicating standard deviation. ND, not detected. Source data

Cloned *dsyGD* conferred in vivo MSM (177.42 ± 3.23 pmol DMSHB per µg of protein per hour) and DDC activity (13.81 ± 0.97 pmol DMSP per µg of protein per hour) when expressed in *E.* *coli* and restored DMSP production in a *L.* *aggregata* LZB033 *dsyB*^*−*^mutant^1^, which does not produce DMSP (Table 1). Furthermore, purified DsyGD (Supplementary Fig. 3a) exhibited in vitro *S*-adenosylmethionine (AdoMet)-dependent MSM and DDC activity with an optimal temperature of 25 °C (Supplementary Fig. 4a) and pH of 7.0 for MSM activity (Supplementary Fig. 4b). Kinetic analysis of DsyGD showed it to have an approximate tenfold higher MSM (*k*_cat_/*K*_m_ of 0.21 and 0.073 µM^−1^ s^−1^ for MTHB and AdoMet, respectively) than DDC catalytic efficiency (*k*_cat_/*K*_m_ of 2.30 × 10^−3^ µM^−1^ s^−1^) (Supplementary Fig. 4c–e). Even with this lower DDC catalytic efficiency, *G.* *sunshinyii* still accumulated 23-fold higher DMSP than DMSHB under standard growth conditions (Fig. 3a).

**Table 1:** Activity assays of the cloned candidate DMSP synthesis genes

Cloned gene	NCBI accession number/MMETSP ID	Candidate DMSP synthesis protein	DMSP or DMSHB production from MTHB in *E.* *coli*	DMSP production from DMSHB in *E.* *coli (*or *R.* *pomeroyi*, Rp*)*	DMSHB and DMSP production from MTHB in the *L.* *aggregata dsyB*^*−*^mutant
Control (host with no cloned gene)	–	–	NT	NT/ND (Rp)	ND
*G.* *sunshinyii* YC6258	WP_044616208.1	^*Gs*^DsyGD	177.42 ± 3.23	13.81 ± 0.97	471.81 ± 24.89
*Symploca* sp. SIO3E6	NES17792.1	DsyGD	83.83 ± 6.24	35.13 ± 1.23	147.31 ± 4.27
*Oscillatoria* sp. SIO1A7	NER39123.1	DsyGD	6.69 ± 0.35	9.16 ± 0.50	93.34 ± 4.27
*Z.* *navalis* LEGE 11467	WP_264320056.1	^*Zn*^DsyG	1.79 ± 0.13	ND	1.96 ± 0.13
Planctomycetales bacterium	MCA9139260.1	DsyG-like	ND	ND	ND
*P.* *umbilicalis*	OSX77567.1	DsyG-like	ND	ND	NT
*Norrisiella sphaerica* BC52	MMETSP0113	Clade A DSYE	2.93 ± 0.12	ND	0.93 ± 0.02
*Bigelowiella longifila* CCMP242	MMETSP1359	Clade A DSYE	1.54 ± 0.05	ND	NT
*O.* *prasinos* BCC99000	MMETSP0933	Clade B DSYE	1.13 ± 0.02	ND	NT
*Tetraselmis striata* LANL1001	MMETSP0803	Clade B DSYE	1.58 ± 0.08	ND	NT
*Pelagococcus subviridis* CCMP1429	MMETSP0882	Clade C DSYE	2.06 ± 0.02	ND	1.92 ± 0.05
*C. mesostigmatica* CCMP1168	MMETSP0047	Clade C DSYE	1.94 ± 0.04	ND	NT
*Pavlova* sp. CCMP459	MMETSP1381	Clade D DSYE	1.19 ± 0.13	ND	NT
*Exanthemachrysis gayraliae* RCC1523	MMETSP1464	Clade D DSYE	1.43 ± 0.07	ND	NT
*F.* *cylindrus* CCMP1102	OEU16654.1	Clade E DSYE	0.54 ± 0.03	ND	1.48 ± 0.13
*N.* *inconspicua*	KAG7362955.1	Clade E DSYE	1.24 ± 0.03	ND	1.35 ± 0.07
*P.* *parvum* Texoma1	MMETSP0008	DsyD-like	NT	ND (Rp)	NT
*A.* *monilatum* CCMP3105	MMETSP0093	DsyD-like	NT	ND (Rp)	NT

Candidate genes were cloned and assayed (*n* = 3 independent biological replicates) for MSM or DDC activity in *E.* *coli* BL21 (DE3) (pET-16b-based clones) or in the *L.* *aggregata dsyB*^*−*^ mutant or *R.* *pomeroyi* DSS-3 (pLMB509-based clones). *R.* *pomeroyi* was used as the host (rather than the *L.* *aggregata dsyB*^*−*^ mutant) for assaying the pLMB509 clones of candidate single-domain DsyDs (from *Prymnesium* and *Alexandrium*), since *R.* *pomeroyi* lacks DDC activity and pLMB509 clones do not express in *E.* *coli*. ND, not detected; NT, not tested. DMSP or DMSHB production units (pmol per µg of protein per hour).

The individual *G.* *sunshinyii* DsyG and DsyD domains and the predicted MTOB reductase (MR) enzyme were either insoluble (for DsyG) and/or did not have the expected MSM, DDC or MR activities (Fig. 1a) when expressed in *E.* *coli* or as purified proteins (Supplementary Fig. 3b–d) under the conditions tested here. It is possible that these specific *G.* *sunshinyii* DsyG and DsyD domains evolved to require each other. Unfortunately, transformation and conjugation of plasmids into *G.* *sunshinyii* were not possible, preventing mutagenic and/or overexpression analysis of DsyGD in this host. Nevertheless, DsyGD is a bifunctional DMSP synthesis enzyme with two DMSP synthesis-specific and sequential enzyme activities in the transamination pathway^50^ and the only known enzyme with DDC activity.

### DsyGD is confined to *G.sunshinyii* and some *Oscillatoriales*

Proteins with a high level of amino acid identity to ^*Gs*^DsyGD were not identified from any other sequenced microbial genomes or transcriptomes. However, proteins with MSM and DDC activity (Table 1) but only an ~46% amino acid identity to ^*Gs*^DsyGD (Supplementary Table 1) were encoded from metagenome-assembled genomes (MAGs) of two *Oscillatoriales* order cyanobacteria (*Symploca* sp. SIO3E6 and *Oscillatoria* sp. SIO1A7) (Figs. 1c and 2). Interestingly, a single-domain DsyG with MSM activity and an ~50% amino acid identity to this domain of ^*Gs*^DsyGD was also identified in *Zarconia navalis* LEGE 11467, an *Oscillatoriales* isolate from a subtidal epilithic marine sample^52^ (Figs. 1c and 2, Supplementary Table 1 and Supplementary Fig. 2). Unlike the truncated ^*Gs*^DsyG, ^*Zn*^DsyG was expressed as a soluble protein in *E.* *coli*, explaining their differences in MSM activity (Supplementary Fig. 3d,e). *Z.* *navalis* lacked *dsyD* and accumulated 111- to 335-fold lower DMSP than DMSHB levels (0.34 ± 0.005 pmol DMSP per µg of protein versus 108.53 ± 8.06 pmol DMSHB per µg of protein in standard conditions; Supplementary Fig. 5a). These data support the hypotheses that the double domain ^*Gs*^DsyGD was responsible for the higher ratio of DMSP:DMSHB in *G.* *sunshinyii* than *Z.* *navalis*, that any *Z.* *navalis* enzyme(s) with DDC activity (currently unidentified) were not efficient or expressed at low levels and that DMSHB may have a more prominent role than DMSP in *Z.* *navalis*.

Unlike DsyG, a single-domain DsyD was not identified from any sequenced genomes, MAGs or transcriptomes. The most homologous proteins to the ^*Gs*^DsyD domain, from *Prymnesium parvum* Texoma1 and *Alexandrium monilatum* CCMP3105, contained only the PF04115.15 domain, showed 34% and 28% amino acid identity to the *Oscillatoria* sp. SIO1A7 DsyD domain and lacked DDC activity (Table 1, Supplementary Tables 1 and 2 and Supplementary Fig. 6). Thus, knowledge on the DDC step of the transamination pathway is still lacking. Note, the unidentified enzymes with DDC activity in DMSP-producing bacteria (such as *L.* *aggregata*), algae and non-DMSP producers (such as *Rhizobium*) are probably more widespread than DsyD^1,15^. DMSHB probably has more important physiological role(s) than DMSP in *Z.* *navalis* and potentially other organisms, inferring that DMSHB may be prominent in marine environments, and that a DDC enzyme is not always required in organisms with MSM activity.

After ^*Zn*^DsyG, the next most homologous proteins to the ^*Gs*^DsyG domain, with an ~39% amino acid identity, were from a *Planctomycetales* bacterium MAG and the red alga *Porphyra umbilicalis* (Supplementary Table 1). These DsyG-like proteins either phylogenetically clustered more closely to the diatom MTHB *S*-methyltransferase (MSM) TpMMT than ^*Gs*^DsyG (*P. umbilicalis*) or were positioned in between TpMMT and ^*Gs*^DsyG (*Planctomycetales* bacterium) (Fig. 2). Note, the *P. umbilicalis* protein, like ^*Gs*^DsyGD, contained two domains, but its C-terminal domain belonged to the aspartate decarboxylase protein family (pfam02261), which seemed a good candidate DDC as a DsyD isoform enzyme. Despite this, both the recombinant *Planctomycetales* and the *P. umbilicalis* DsyG-like proteins lacked MSM and DDC activity (Table 1). There were also no proteins with high homology to DsyG or DsyD (>38% or 29% amino acid identity, respectively) predicted from the genomes and/or transcriptomes of eukaryotic algae. Overall, these data support *dsyGD*/*dsyG* as reliable indicators for DMSP/DMSHB synthesis in bacteria and filamentous cyanobacteria not previously suspected to produce these molecules. These data also highlight the need for careful functional analysis of DMSP synthesis genes and enzymes before predicting DMSP synthesis in organisms based on their presence. This is particularly relevant for TpMMT, which has only been characterized from *T.* *pseudonana*^16^.

### Regulation of DMSP production in *Gynuella* and *Zarconia*

In *G.* *sunshinyii*, DMSP and DMSHB accumulation and ^*Gs*^*dsyGD* gene transcription were significantly upregulated by growth in media with increased salinity or decreased nitrogen levels, with DMSP and DMSHB either low or undetected under low salinity or high nitrogen conditions (Fig. 3a,b and Supplementary Fig. 7a,b). Note, *G.* *sunshinyii* accumulated nitrogenous glycine betaine (GB) as a probable major osmolyte, whose levels always far exceeded DMSP/DMSHB, except under low salinity conditions, where both GB and DMSP/DMSHB were undetected (Supplementary Fig. 7a,b). *Z.* *navalis* also accumulated more DMSHB (and ^*Zn*^*dsyG* transcripts) with increased salinity and showed reduced levels in high nitrogen conditions (Supplementary Fig. 5a,c). GB production was higher than DMSP/DMSHB (Supplementary Fig. 5b,d), indicating that this may also be a major osmolyte in *Z.* *navalis*. In contrast, DMSP accumulated to comparatively very low and constitutive levels in *Z.* *navalis* irrespective of the growth conditions (Supplementary Fig. 5a). These data are consistent with findings on other DMSP-producing organisms^14,15,49^, where DMSP and/or DMSHB potentially act as sulfur osmolytes, whose production over nitrogen-containing equivalents may be advantageous in sulfur-rich but nitrogen-sparse marine settings, and expression of any unknown *Z.* *navalis* DDC enzyme(s) either being very low and/or not regulated by salinity or nitrogen levels. Note, DMSHB and DMSP production also releases nitrogen from the transamination of Met (Fig. 1).

Further supporting the role of DMSP and DsyGD in osmoprotection, cloned ^*Gs*^*dsyGD* greatly enhanced the growth of an osmosensitive *E.* *coli* strain FF4169 (ref. ^53^) under increased salinity in the presence of MTHB (which has limited osmoprotective properties^50^) or, especially, DMSHB, compared with control strains lacking cloned *dsyGD* (Fig. 3c). This osmoprotection phenotype was probably due to DMSHB and/or DMSP produced from MTHB and DMSHB (5.49 ± 0.99 and 10.13 ± 0.63 pmol DMSP per µg protein per hour, respectively), since *E.* *coli* strain FF4169 lacking cloned ^*Gs*^*dsyGD* produced no DMSP from MTHB or DMSHB (Fig. 3d). Although this work was conducted in *E.* *coli* and not a marine organism, it demonstrates that cloned DMSP synthesis genes can confer osmoprotection, which may be of importance for biotechnological applications.

### Identification of DSYE in diverse algae

Although no DsyGD proteins were predicted in eukaryotic algae, single-domain DsyG-like proteins were identified with <38% amino acid identity to ^*Gs*^DsyG from sequenced algal genomes (*Fragilariopsis cylindrus* CCMP1102 and *Nitzschia inconspicua* strain hildebrandi). Furthermore, 61 DsyG-like proteins were predicted from the 397 different marine eukaryotes in the Marine Microbial Eukaryote Transcriptome Sequencing Project (MMETSP) database^54^ (Supplementary Table3). These algal proteins, termed DSYE (‘E’ for eukaryotes and DSY in upper case to denote their eukaryotic host), were phylogenetically distinct to cyano/bacterial DsyG and were themselves divided into five separate clades (termed DSYE clade A–E) (Fig. 2). Multiple representative DSYE proteins from the five clades were expressed in *E.* *coli* and all showed MSM activity (Table 1 and Fig. 2).

Clade A DSYE proteins were identified in *Chloroarachniophyta*, notably *Bigelowiella natans*, which are known to accumulate high levels of DMSP^13^ and *Norrisiella* spp., which are not previously known to produce DMSP (Table 1 and Fig. 2).

Clade B DSYE proteins were in diverse and highly abundant chlorophyte algae, including *Tetraselmis* sp.^55^, *Pyraminonas* sp.^55^, *Bathycoccus* sp.^56^ and *Mantoniella* sp.^55^ (which are known to accumulate low levels of DMSP); *Micromonas* sp. (which contain both high and low DMSP-producing representatives^55,56^) and *Ostreococcus* sp. (a widely distributed genus in Earth’s oceans^57^ not previously known to produce DMSP) (Fig. 2 and Supplementary Table 3). Tested *Ostreococcus tauri* cells contained DMSP (0.34 ± 0.003 nmol DMSP per µg of protein), consistent with members of this genus being DMSP producers (Supplementary Table 4).

Clade C DSYE proteins were mostly in pelagophyte algae, for example, *Pelagococcus* sp., such as *P.* *subviridis* CCMP1429, which had DSYE and DSYB^15^, and *Pelagomonas* spp., both thought to accumulate low levels of DMSP^13,55,56^ (Fig. 2). Pelagophyte algae were not thought to be globally important DMSP producers, and few had been studied for DMSP production, despite these picoeukaryotes often forming large blooms and being globally abundant^58–61^. Here, diverse axenic bloom-forming and sometimes toxin-producing pelagophytes^58–60^ *Chrysocystis*, *Aureococcus*, *Pelagococcus*, *Chrysoreinhardia* and *Pelagomonas* strains were shown to accumulate DMSP to intracellular concentrations ranging from 13.79 ± 0.46 to 233.81 ± 32.10 mM, (Supplementary Table 4 and Supplementary Fig. 8). Thus, pelagophytes, for example, *Pelagomonas calceolata*, one of the most abundant eukaryotic species in Earth’s oceans^61^, are potentially important global DMSP producers.

Haptophytes are generally thought to accumulate high DMSP levels and contain *DSYB*^15,62^. *Pavlova* spp. and *Exanthemachysis* spp. are exceptions that lack *DSYB* but contain a functional clade D *DSYE* (Fig. 2 and Supplementary Table 3). Most *Pavlova* spp. are high DMSP accumulators, but some, for example, *P.* *lutheri*, are considered low DMSP accumulators, as are all tested *Exanthemachysis* spp.^13^.

Clade E DSYE proteins were exclusively in diatoms, generally thought to accumulate low intracellular DMSP levels^13,55^. None of the diatoms with DSYE contained TpMMT, although some did also contain *DSYB*, for example, *F.* *cylindrus* CCMP1102 and *Pseudonitzschia fraudulenta* WWA7, while others, for example, *N.* *inconspicua*^15^, contained only *DSYE* (Fig. 2 and Supplementary Table 3).

Purified clade B and C DSYE from *Ostreococcus prasinos* BCC99000 and *Chroomonas mesostigmatica* CCMP1168 (Table 1 and Supplementary Fig. 9) showed in vitro AdoMet-dependent MSM activity with temperature and pH optima of 30 °C and 20 °C (Supplementary Fig. 10a,e) and 9.0 and 9.5 (Supplementary Fig. 10b,f), respectively. The *C.* *mesostigmatica* clade C DSYE was ~30-fold more efficient with MTHB (*k*_cat_/*K*_m_ of 4.5 × 10^−3^ μM^−1^ s^−1^) than the *O.* *prasinos* clade B DSYE enzyme (*k*_cat_/*K*_m_ of 0.15 × 10^−3^ μM^−1^ s^−1^) (Supplementary Fig. 10). Note, these DSYE enzymes were 40- and 1,400-fold, respectively, less efficient than ^*Gs*^DsyGD. Further work is required to establish whether DSYE catalytic efficiency and/or its expression levels are robust reporters of the DMSP levels that organisms accumulate.

Identification of *DSYE*, in addition to *DSYB* and *TpMMT* in algae and *dsyGD*, *dsyG*, *dsyB* and *mmtN* in diverse bacteria, has greatly expanded the ability to predict which organisms, particularly algae, can produce DMSP (Fig. 2 and Supplementary Table 3). With the inclusion of *DSYE*, 66% of the predicted 162 DMSP-producing eukaryotes^13^ within MMETSP expressed a known *S*-methyltransferase gene involved in DMSP synthesis, an increase from 44% when considering only DSYB and TpMMT (Supplementary Table 3). Most of the remaining candidate DMSP producers on MMETSP which lacked *DSYE*, *DSYB* or *TpMMT* had not been tested for DMSP production or were predicted to accumulate low DMSP levels (Supplementary Table 3). Outside of MMETSP data, there are still known DMSP-producing organisms which lack any of these *S*-methyltransferase genes, but their numbers are now reduced and are mainly confined to plants such as *Spartina* spp. and *Melanthera biflora* that utilize the methylation pathway for DMSP synthesis^31,63^, macroalgae, such as *Ulva* spp., and cyanobacteria such as *Trichodesmium* that accumulate low DMSP levels^64^.

### Algae containing DSYE are abundant in Earth’s oceans

The Ocean Microbial Reference Gene Catalogue (OM-RGC_V2) metagenomic dataset^65^, generated from 0.22–3 µm fractionated samples and apportioned to bacterioplankton, was analysed for known DMSP synthesis genes. As previously described, *dsyB* and its transcripts were far more abundant than those for *mmtN* in Earth’s oceans, and these *dsyB* genes/transcripts were over twofold more abundant in the surface (SRF) and deep chlorophyll maximum (DCM) than in mesopelagic (MES) waters (Supplementary Fig. 11, Supplementary Fig. 12 and Supplementary Table 5). *dsyGD/dsyG* genes and transcripts were not detected in any OM-RGC_V2 dataset, consistent with this system being largely irrelevant to marine DMSP cycling. Alternatively, some bacteria, notably filamentous cyanobacteria, containing these genes, may have aggregated and not been captured by the bacterioplankton sampling methods. However, eukaryotic *DSYE* clade B genes and transcripts from chlorophyte algae (picoeukaryotes including *Pyramimonas*, *Pterosperma*, *Ostreococcus*, *Micromonas* and *Tetraselmis*), small enough to be in the bacterioplankton samples, were present in almost all stations, at approximately twofold lower levels than *dsyB* in SRF and DCM samples (Supplementary Fig. 12). Approximately 6% of the picoeukaryotes in these SRF and DCM samples contained *DSYE*. Consistent with the phototrophic lifestyle of their algal hosts, *DSYE* and its transcripts were barely detected in MES samples (Supplementary Table 5 and Supplementary Fig. 11). OM-RGC_V2 *DSYE* and *dsyB* genes and transcripts were most abundant in high-latitude polar samples, with a few exceptions. Notably, maximal *dsyB* abundance was seen in a mid-latitude DCM sample (Supplementary Fig. 12).

Within the eukaryotic Marine Atlas of Tara Ocean Unigenes (MATOU), algal DMSP synthesis genes and transcripts were also barely detected in data from MES but were much better represented in the SRF and DCM samples, consistent with their presence in phototrophs (Supplementary Table 6). Although *DSYB* genes, mostly from haptophytes and dinophytes, were detected in all stations, *DSYE* genes, predominantly from pelagophytes (clade C) and to a lesser extent, chlorophytes (clade B), were marginally and approximately twofold more abundant in the photic SRF and DCM samples, respectively (Supplementary Figs. 11 and 12 and Supplementary Table 5). The *DSYB* and *DSYE* genes showed similar biogeographical distribution patterns in MATOU stations, being concentrated in non-polar sites between −50° and 50° latitude (Supplementary Fig. 12). In contrast to the metagenomic data, *DSYB* transcripts were approximately twofold more abundant than those for *DSYE* in SRF and DCM samples from MATOU datasets (Supplementary Fig. 11 and Supplementary Table 5), and this may be a better indication of DMSP production than gene abundance. Diatom *TpMMT* and their transcripts were generally one to two orders of magnitude less abundant than those for algal *DSYB* or *DSYE* (Supplementary Fig. 11 and Supplementary Table 5). These data are consistent with previous reports of haptophytes and dinophytes^15^, and also now pelagophyte algae, being important global DMSP producers, with most diatoms having a less prominent role. Further model organism and environmental sampling work on diverse pelagophyte algae is required to explore their importance in global DMSP cycling, especially during blooms^66^, where they are likely to have a more considerable impact.

## Discussion

**Fig. 4:**
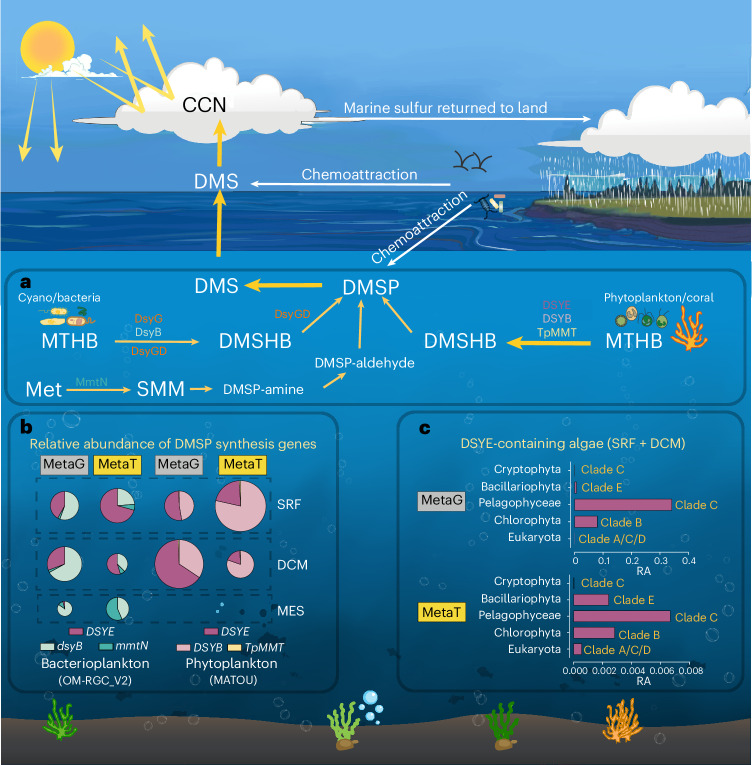
Overview of key DMSP biosynthesis enzymes and pathways and their environmental importance. **a**, Key DMSP synthesis and cleavage pathways are indicated with known algal and bacterial *S*-methyltransferases. **b**, The relative abundance of DMSP synthesis genes and transcripts in SRF, DCM and MES water layers from OM-RGC_V2 (0.22–3 µm size fraction) and MATOU (0.8–2,000 µm size fraction) datasets. **c**, Clades and taxonomy of *DSYE* sequences detected in MATOU datasets. The genes in OM-RGC_V2 and MATOU datasets were normalized to *recA* and *β-actin* genes, respectively. The size of the pie charts represents the gene relative abundance in the corresponding datasets. Note, no *dsyG*/*dsyGD* sequences were detected. CCN, cloud condensation nuclei. MetaG, metagenomes data; MetaT, metatranscriptomes data; RA, relative abundance. SMM, *S*-methylmethionine.

DMSP is an abundant and ecologically important marine organosulfur compound. This study identifies the unusual DMSP synthesis genes *dsyGD*/*dsyG* in the rhizobacterium *G. sunshinyii* and filamentous cyanobacteria, not previously suspected to produce DMSP (Fig. 4), and provides evidence for DMSP and/or DMSHB being osmolytes in these bacteria. The origin and transfer of *dsyG*/*dsyGD* between organisms was interesting but difficult to address because these genes were rare in sequenced organisms and environmental samples.

Functional genomics identified DSYE, forming a diverse family of eukaryotic MSM enzymes that were phylogenetically distinct from DsyG and other known enzymes with MSM activity. The five DSYE clades (A–E) comprised taxonomically distinct eukaryotic algae spanning low, medium and high DMSP accumulators, and algae not previously reported to produce DMSP (for example, *O.* *tauri*) and multiple pelagophyte algae. *DSYE*, with *DSYB* and *TpMMT*, serve as indicator genes of DMSP synthesis, and their combined presence in most known DMSP-producing algae with available transcriptomic and genomic data, allows more comprehensive predictions of key algal producers in marine environments with available multi-omics data.

A major unanswered question was whether the presence of a particular DMSP synthesis gene implies how much DMSP an organism accumulates. McParland et al. suggested that the presence of *DSYB* or *TpMMT* in algae was a reporter of high or low DMSP accumulation levels, respectively^62^. This appealing hypothesis was supported by a strong correlation between *DSYB* and high DMSP accumulators (Supplementary Table 3)^13^. However, the bacterial DsyB enzyme is as efficient as algal DSYB, despite bacteria generally accumulating low intracellular levels of DMSP^13^, and there are many examples of organisms with *DSYB* that also accumulate low intracellular DMSP levels (for example, *F.* *cylindrus* and *Chrysochromulina tobin*^15^). It is more difficult to infer the reverse correlation for TpMMT because this protein has only been studied in *T.* *pseudonana*^16^. However, all proteins with high homology to *T.* *pseudonana* TpMMT were from diatoms, predicted to accumulate low DMSP levels (Supplementary Table 3), supporting this notion. Considering *DSYE* was found in organisms predicted to be both low and high DMSP accumulators^13^, it would be difficult to predict an organism’s intracellular DMSP level based on *DSYE* occurrence (Supplementary Table 3), exemplified by the varied DMSP levels seen in pelagophyte algae with *DSYE*. Previous research has shown that gene transcript and protein levels were more robust indicators of an organism’s potential DMSP levels^13^, since these are guided by varying environmental conditions, for example, nitrogen and salinity levels, and govern DMSP synthesis potential, along with substrate availability. Finally, most studies only report the intracellular DMSP levels in producers, which is affected by both DMSP production and turnover, and DMSP production will be dependent on any variability in growth conditions. Therefore, although the different factors discussed here may give clues or indicate gross trends in DMSP production, prediction of a particular organism’s DMSP content is difficult in the absence of direct measurement.

The *dsyGD*/*dsyG* and *DSYE* genes were at different ends of the spectrum for their perceived importance in marine environments. Bacteria with *dsyGD*/*dsyG* were not detected in any TARA metagenomic or metatranscriptomic dataset, consistent with them having a negligible role in marine DMSP production. Furthermore, *dsyGD*/*dsyG* could not be detected in metagenomic data from *Spartina* rhizosphere samples in which *G.* *sunshinyii* was present^67^, suggesting that *dsyGD* may not even be universal in this species. In contrast, *DSYE* genes, particularly from pelagophyte and chlorophyte algae, were more abundant than *DSYB* (largely from haptophytes and dinophytes) and orders of magnitude more abundant than *TpMMT* from diatoms in Earth’s SRF waters. However, *DSYE* transcripts were approximately twofold less abundant than *DSYB* transcripts in these samples, which is probably a better reporter of DMSP production. Even with these reduced transcript levels, pelagophyte and chlorophyte algae with *DSYE* should be considered as potentially important marine DMSP producers, especially given that many of these algae form large blooms, are globally abundant^66^ and were shown here to accumulate medium to high levels of DMSP. Further work on these algae in the natural environment is vital because they have not received the same attention from DMSP biologists as, for example, haptophyte and dinophyte algae^68,69^.

Assuming that the known *S*-methyltransferase genes in microbial DMSP synthesis pathways were the major isoforms, which our analysis of algal transcriptomes implied, it was puzzling why these genes and their transcripts were not more abundant in marine systems. This is an especially relevant question considering the marine ubiquity of DMSP and DMSP catabolic genes, for example, *dddP*, predicted to be in 5.29% of SRF marine bacteria^70^. There are still many DMSP producers that lack known DMSP synthesis genes, for example, DMSP-producing plants, macroalgae such as *Ulva* spp., cyanobacteria such as *Trichodesmium* and *Synechococcus* and other bacteria, for example, *Marinobacter* sp.^14^, but these are not expected to be major DMSP producers on the same scale as haptophyte, dinophyte and now pelagophyte algae, for instance. It is possible that these phototrophs contain other unidentified isoform MSM enzymes or DMSP synthesis pathways with unknown enzymes. This was proposed for the dinophyte *Crypthecodinium cohnii*, which has multiple *DSYB* copies^15^ but is thought to utilize a Met decarboxylation pathway^10,12^, for which no genes or enzymes are known. Finally, it is also possible that the DMSP synthesis gene products are more abundant and active than their gene and transcript abundance implies. Further molecular work is required on model marine organisms to address these important questions, combined with more comprehensive environmental quantification of DMSP stocks, synthesis and catabolism rates and of DMSP biosynthetic enzyme abundance.

## Methods

### Strains, plasmids and culture conditions

Strains, plasmids and primers used in this study are listed in Supplementary Tables 7, 8 and 9. *G.* *sunshinyii*, *L.* *aggregata dsyB*^*−*^ mutant strain and *R.* *pomeroyi* DSS-3 were grown in YTSS (yeast tryptone sea salts)^71^ or MBM minimal medium^72^ (10 mM succinate carbon source, 10 mM NH_4_Cl nitrogen source and 35 practical salinity units (PSU)) at 30 °C. Where indicated, MBM salinity and/or nitrogen content was adjusted by altering the amount of sea salts (Merck; S9883) or NH_4_Cl added, respectively. *Z.* *navalis* LEGE 11467 was grown in BG-11 medium^73^ supplemented with varying amounts of sea salts and NaNO_3_ at 22 °C under 12 h light (50 μmol photons per square metre per second)/12 h dark cycles with 170 rpm shaking. *E.* *coli* strains were grown in lysogeny broth (LB) or M9 minimal medium^74^ at 37 °C. *R.* *leguminosarum* J391 was grown in TY or Y medium at 28 °C (ref. ^75^) with 180 rpm shaking. All eukaryotic algae were grown in F/2 medium with 16 h light (50 μmol photons per square metre per second)/8 h dark cycles, as in Curson et al.^15^. Where necessary, algal medium was modified according to the requirements of the experimental conditions being tested. All liquid cultures were grown with shaking at 180–200 rpm, unless specified otherwise. Where necessary, antibiotics were added to media at the final concentrations specified as follows: ampicillin 100 µg ml^−1^, streptomycin 400 µg ml^−1^, kanamycin 20 µg ml^−1^, rifampicin 20 µg ml^−1^, tetracycline 10 µg ml^−1^ and gentamicin 20 µg ml^−1^ (or 80 µg ml^−1^ for *L.* *aggregata dsyB*^*−*^ mutant with pLMB509).

### Quantification of MeSH, DMS, DMSHB and DMSP

Gas chromatography (GC) was the primary method used to quantify DMSP and DMSHB. All GC assays involved measurement of either headspace MeSH, as described in Carrión et al.^76^ or of DMS (either produced directly or through alkaline lysis of DMSP and/or DMSHB), as described in Curson et al.^15^ for culture-dependent and protein work or as in Williams et al.^14^ for work on environmental samples. These assays were conducted using a flame photometric detector (Agilent 7890 A GC equipped with a 7693 autosampler) along with a capillary column (HPINNOWax 30 m × 0.320 mm, Agilent Technologies J&W Scientific). The detection limit for headspace DMS was 0.0067 µM DMSP and DMSHB in water and media respectively and 1 µM DMSP in methanol; MeSH was 27 µM in water/media.

### DMSP content in *C.scabrifolia*

*C.* *scabrifolia* plants and rhizosphere soil were obtained in a saltern area in Shandong Province, China (120.745° E, 36.454° N). *C.* *scabrifolia* plants were carefully uprooted and placed into sterile plastic bags. The plant material was washed to remove sediment and separated into different tissue types (roots and leaves) using ethanol sterilized scissors or tweezers and assayed for DMSP. The *C.* *scabrifolia* rhizosphere was sampled, as in Williams et al.^14^. Briefly, 5 g roots were sampled, and rhizosphere was subjected to vortexing five times to collect the adhered soil. The samples were assayed for DMSP by GC as above and normalized to wet weight.

### DMSP synthesis in *G.sunshinyii*

To infer the *G.* *sunshinyii* DMSP synthesis pathway, the cultures were incubated overnight in YTSS, adjusted to an OD_600_ of 0.3 and washed three times with 35 PSU MBM. The samples were then diluted 1:100 into 5 ml 35 PSU MBM with or without (control) 0.5 mM DMSP synthesis intermediates (l-Met (Merck, M9625), MTOB (Merck, K6000), MTHB (Merck, 55875), DMSHB, DMSP-amine, 3-methylthiopropylamine (Merck, 639095), methylmercaptopropionate (Tokyo Chemical Industry, M0811) and incubated for 24 h at 30 °C. DMSHB and DMSP-amine were synthesized, as in Curson et al.^1^. Apart from l-Met, all chiral DMSP intermediates were thought to be a 50:50 mixture of d- and l-forms.

To study DMSHB/DMSP accumulation in *G.* *sunshinyii* under varied environmental conditions, the cultures were grown under standard (35 PSU, 0.5 mM NH_4_Cl), low salinity (5 PSU, 0.5 mM NH_4_Cl), high salinity (50 PSU, 0.5 mM NH_4_Cl) and high nitrogen (35 PSU, 10 mM NH_4_Cl) conditions. *G.* *sunshinyii* was inoculated into 50 ml YTSS and incubated with shaking at 30 °C overnight. The cultures were then washed three times by centrifuging at 17,000*g* for 5 min and resuspending in 35 PSU MBM without nitrogen added. A total of 1 ml of washed cells was then inoculated into 10 ml MBM as described for the different conditions and incubated at 30 °C for 24 h. Three biological replicates were prepared for each condition, and DMSP amounts were normalized to protein concentrations determined using the Bradford method, as in Curson et al.^1^.

To quantify in vitro MSM and DDC activities in *G.* *sunshinyii*, 5 ml YTSS overnight cultures were collected by centrifugation at 17,000*g* for 5 min, washed three times with 1 ml 50 mM Tris–HCl buffer (pH 7.5) and then resuspended in 1 ml 50 mM Tris–HCl buffer. Subsequently, the cells were sonicated (3 × 10 s) on ice using a Markson GE50 Ultrasonic Processor set to an output of 70, then centrifuged at 17,000*g* for 5 min to pellet the debris. The resultant supernatants (cell-free extracts) were dialysed to remove any pre-existing metabolites, using dialysis tubing (3,500 Da molecular weight cut off; Spectrum Labs) in 2 l of dialysis buffer (20 mM HEPES, 150 mM NaCl, pH 7.5) at 4 °C overnight^15^. A total of 200 µl of cell-free extracts with nothing added (control) or with 1 mM MTHB plus 1 mM AdoMet (New England Biolabs, B9003S) or just 1 mM DMSHB were placed into GC vials and incubated at 30 °C for 30 min. After incubation, 100 µl 10 M NaOH was added to cell-free extracts and assayed for DMSHB and/or DMSP by GC, as above.

### Prediction of *G.sunshinyii* DMSP synthesis and catabolic genes

The *G.* *sunshinyii* genome sequence and protein annotation data were downloaded from the National Center for Biotechnology Information (NCBI) (PRJNA233633) and searched for DMSP synthesis and catabolic proteins using local BLASTp and verified probe sequences (Supplementary Table 10) with an *E*-value threshold of ≤1 × 10^−5^, amino acid identity of ≥40% and coverage of ≥70%.

### Screening of *G.sunshinyii* genomic library

A *G.* *sunshinyii* genomic library was constructed in the cosmid pLAFR3 (ref. ^77^), as described in Curson et al.^45^. Briefly, 2.5 µg of *G.* *sunshinyii* high-quality genomic DNA was partially digested with *Eco*RI, followed by ligation into 1.0 µg of pLAFR3 cosmid DNA that had been fully digested with *Eco*RI and dephosphorylated. Subsequently, 0.7 µg of ligated DNA was packaged into recombinant *λ* phage using Gigapack III XL packaging extracts (Agilent Technologies, 200209). The packaged DNA was then transfected into *E.* *coli* 803 to produce the *G.* *sunshinyii* genomic library. The library comprising 90,000 clones was transferred en masse into the heterologous host *R.* *leguminosarum* J391 by conjugation using an *E.* *coli* helper strain containing the plasmid pRK2013 (ref. ^78^). The transconjugants were inoculated into 200 µl Y medium containing 0.5 mM MTHB in 2 ml GC vials, incubated at 30 °C for 48 h and assayed for DMSHB and DMSP by GC analysis as above. The DMSHB and DMSP levels in the headspace were normalized to protein levels, as above. *R.* *leguminosarum* J391 with empty pLAFR3 cosmid and media only, with and without MTHB substrate, were used as controls. J391 has DDC activity, so any DMSHB produced through MSM activity would lead to DMSP production^1^.

### Osmotolerance experiments in *E.coli* strains

*E.* *coli* strain MC4100 and FF4169 (*otsA*^−^)^50,53^ (Supplementary Table 7) were used to study osmotolerance conferred by cloned ^*Gs*^*dsyGD*. The ^*Gs*^*dsyGD* gene and its promoter region was synthesized and cloned in pUCm-T (by Sangon Biotech, Shanghai Co., Ltd.; Supplementary Table 8) to make pJDT0029 and transformed into *E.* *coli* FF4169. The *E.* *coli* strains MC4100, FF4169 and FF4169:pJDT0029 were grown in LB medium overnight (in triplicate). All starter cultures were adjusted to an OD_600_ of 0.3 and washed twice with M63 medium lacking NaCl and sulfur, followed by resuspension in 1 ml M63, as in Summers et al.^50^. The suspensions were diluted 1:100 in fresh M63 medium (22 mM d-glucose as carbon source and 1 mM MgSO_4_ as sulfur source) with high salinity (0.5 M NaCl) and DMSP, GB, MTHB or DMSHB at 1 mM final concentration. A total of 0.1 mM IPTG was added to induce expression of ^*Gs*^*dsyGD* from pJDT0029 in FF4169. The growth was monitored by measuring OD_600_ using a plate reader (Thermo Scientific, Multiskan GO) every 1 h until stationary phase.The DMSP production was confirmed by GC at the end of each experiment.

### Identification of DsyGD, DsyG, DsyD and DSYE homologues

The prokaryotic ^*Gs*^DsyGD, ^*Gs*^DsyG and ^*Gs*^DsyD homologues in the NCBI were identified by BLASTp using an *E*-value cut-off of 1 × 10^−55^ and 38–50% amino acid identity (Supplementary Table 1). To identify eukaryotic DSYE and DsyD-like enzymes BLASTP searches (*E*-value of 1 × 10^−55^ and ≥70% coverage for ^*Gs*^DsyG and *E*-value of 1 × 10^−5^ for DsyD domains) were performed against the predicted proteomes from genomes on the NCBI and the 678 transcriptomes available at MMETSP^54^ (Supplementary Tables 1 and 2).

### Growth of *Z.navalis* under different conditions

*Z.* *navalis* LEGE 11467 (ref^. 52^) was obtained from the Blue Biotechnology and Ecotoxicology Culture Collection (LEGE-CC) from CIIMAR in Portugal and grown with shaking at 22 °C in 50 ml BG-11 medium at 25 PSU (with 0.5 mM NaNO_3_ as the nitrogen source), unless otherwise stated, as described in Rippka et al.^73^. Note that *Z.* *navalis* grows as a floating mass or masses in liquid culture. The triplicate samples were then set up by introducing 100 mg of *Z.* *navalis* material into 25 ml BG-11 medium with different salinities or nitrate concentrations as follows: standard conditions (25 PSU, 0.5 mM NaNO_3_), low salinity (5 PSU, 0.5 mM NaNO_3_), high salinity (50 PSU, 0.5 mM NaNO_3_) and high nitrogen (25 PSU, 17.65 mM NaNO_3_). The samples were taken 14 days after inoculation by removing *Z.* *navalis* material with sterile forceps to 1.5 ml centrifuge tubes, and the wet weight of material (after removing any residual liquid by pipette) was recorded. The samples were stored at −80 °C until GC and/or nuclear magnetic resonance (NMR) analysis. DMSP, DMSHB or GB amounts were normalized to micrograms of protein (determined by Bradford assay as above).

### Quantification of DMSP in *Pelagophyceae* algae

Cultures of *Pelagophyceae* algae (Supplementary Table 7) were incubated for 20 days at 22 °C under 16 h light (120 µmol photons per square metre per second)/8 h dark cycles. Subsequently, 4 ml of culture were centrifuged at 6,000*g* for 10 min, and the pellet was resuspended in 200 µl methanol. The samples were stored at −20 °C for 24 h to allow for extraction of cellular metabolites. The methanol extracts were transferred to GC vials and 100 µl 10 M NaOH was added. The vials were immediately sealed and incubated at 22 °C for 24 h in the dark before DMSP measurements by GC. All experiments were performed in triplicate. The cell numbers in the cultures were quantified using a using a CASY model TT cell counter (Sedna Scientific).

### NMR analysis of DMSP, DMSHB and GB

NMR was used to confirm the presence of DMSP/DMSHB and GB in cyano/bacteria and algae and to estimate the concentration and relative levels of these osmolytes. *G.* *sunshinyii, Z.* *navalis* LEGE 11467 and *Pelagophyceae* algae cultures grown under the conditions described in their corresponding sections were spun down, and the cell pellets were resuspended in 800 μl of deuterium oxide (D_2_O, Merck; 113366). The samples were then transferred to 2 ml tubes containing 0.1–1.4 mm beads and homogenized using the FastPrep-24 5 G (FP5G, FastPrep system, MP Biomedicals) for three cycles of 40 s at 6.0 m s^−1^. The samples were centrifuged at 5,000*g* for 10 min at 4 °C. Subsequently, pyrazine (Sigma-Aldrich) was added at 1 mM final concentration to 500 µl supernatants as internal standard before NMR analysis. The NMR experiments were performed, as in Carrión et al.^70^, using a double echo excitation sculpting component for water suppression (Bruker library zgesgp) and 2 ms Sinc shaped pulses, 128 scans, relaxation delay of 1 s and acquisition delay of 2 s. All spectra were phased, base-corrected and calibrated for the pyrazine peak at 8.64 ppm. The chemical shift of the methyl groups of GB ((CH_3_)_3_N) was at 3.26 ppm (298 K). The methyl groups of DMSP and DMSHB ((CH_3_)_2_S) overlap at 2.91 ppm (298 K); therefore, it was not possible to distinguish them at low concentrations by NMR. Thus, the singlet at 2.91 ppm was taken as the sum of the DMSP and DMSHB concentrations (and refer to them as ‘DMSP/DMSHB’ thereinafter). The GB and DMSP/DMSHB concentrations were estimated by using the following equation:

$$\left[A\right]=\frac{{I}_{A}}{{I}_{P}}\times\frac{{N}_{P}}{{N}_{A}}\times [P],$$

where [*A*] is the molar concentration of the analyte, *I* is the absolute integral of either the analyte (*A*) or pyrazine (*P*), *N* is the number of nuclei corresponding to the peak (*N* = 4 for pyrazine, *N* = 9 for GB and *N* = 6 for DMSHB/DMSP) and [*P*] is the pyrazine molar concentration. These absolute concentrations were then multiplied by the dilution factor derived from manipulation of the initial culture to the NMR tube, divided by the correction factors derived from the calibration curves (2.96 for GB and 2.72 for DMSP/DMSHB) and normalized to cell volume or micrograms of protein. The calibration curves for GB and DMSP/DMSHB were performed using 0.2–1.6 mM standards and 1 mM pyrazine and plotted to obtain straight lines with *R*^2^ of 0.99, where the obtained slope was used as the correction factor. The detection limits for GB and DMSP/DMSHB were 10 and 15 µM, respectively. The DMSP/DMSHB concentrations in *Z.* *narvalis* samples were below the detection limit; therefore, only estimation of GB levels was possible in these samples.

### RNA isolation and RT–qPCR assays

*G.* *sunshinyii* was cultured in triplicate under the conditions described in the ‘DMSP synthesis in *G.* *sunshinyii*’ section above. *Z.* *navalis* LEGE 11467 starter cultures were grown as in ‘Growth of *Z.* *navalis* under different conditions’ then inoculated to 50 ml BG-11 medium with 17.65 mM NaNO_3_ and different salinities for standard (25 PSU), low (5 PSU) and high salinity (50 PSU) and sampled after 14 days. The cell pellets were stored at −80 °C with RNAlater RNA stabilization reagent (Qiagen; 76104) before RNA extraction.

Total RNA from *G.* *sunshinyii* and *Z.* *navalis* LEGE 11467 cultures was extracted using a Direct-zol RNA Miniprep kit (Zymo Research; R2050) and reverse transcribed with a QuantiTect Reverse Transcription Kit (Qiagen; 205311) following the manufacturer’s instructions. Quantitiative polymerase chain reaction with reverse transcritption (RT–qPCR) assays were performed in triplicate with primers listed in Supplementary Table 9 on an AriaMx Real-Time PCR system (Agilent) using a QuantiTect SYBR Green PCR Kit (Qiagen; 204343) and the following cycling conditions: 95 °C for 3 min, 40 cycles of 95 °C for 20 s, 60 °C for 30 s and 72 °C for 30 s.

### In vivo MSM, DDC and MR enzyme assays

Full-length *G.* *sunshinyii dsyGD* (including the *dsyG* methyltransferase and *dsyD* decarboxylase domains), the separate *dsyG* and *dsyD* domain genes and the putative reductase gene were PCR-amplified and cloned into pET-22b (Supplementary Tables 8 and 9). The individual ^*Gs*^*dsyG* and ^*Gs*^*dsyD* domain sequences were determined from their homology to Pfam domains (https://www.ebi.ac.uk/interpro/) and to the functional ^*Zn*^*dsyG* (Supplementary Fig. 2). An existing ATG start codon, corresponding to the penultimate codon of ^*Zn*^*dsyG*, was used to initiate the ^*Gs*^*dsyD* domain. For ^*Gs*^*dsyG*, a stop codon was introduced immediately before this ATG codon. The homologous *dsyGD, dsyG, dsyD* and *DSYE* genes were synthesized and cloned into pET-16b or pET-22b (by Sangon Biotech, Shanghai Co., Ltd.; Supplementary Table 8). All the clones were verified by sequencing and transformed into *E.* *coli* BL21 (DE3). The transformants were cultured in LB containing ampicillin at 37 °C to an OD_600_ of 0.8–1.0 and then incubated at 18 °C for 14 h with 0.1–0.4 mM IPTG for in vivo enzyme assays and protein purification work (see the ‘Protein purification’ section). These cells were either incubated with 0.5 mM MTHB or DMSHB and assayed for in vivo MSM or DDC activity (by GC, as above), respectively, or with nothing added for control experiments. Except for ^*Gs*^DsyG, all tested proteins overexpressed in *E.* *coli* were seen in the soluble fraction in SDS–polyacrylamide gel electrophoresis analysis (Supplementary Figs. 3 and 9).

To further study the in vivo MSM and DDC activity of *dsyGD*, *dsyG*, *dsyD*, *DSYE* and homologous genes, these were cloned into the wide host range taurine-inducible expression plasmid pLMB509 (ref. ^79^) (Supplementary Table 8). These plasmids were conjugated into the *L.* *aggregata dsyB*^*−*^mutant, which makes no DMSP and/or *R.* *pomeroyi* DSS-3 (for *dsyD* clones as it cannot produce DMSP from DMSHB^1^) using the helper plasmid pRK2013 (ref. ^78^), as described in Curson et al.^1^. For MSM and DDC activity assays, triplicate cultures were grown in YTSS at 30 °C for 24 h. The cultures were then adjusted to an OD_600_ of 0.3, washed three times with 35 PSU MBM and diluted 1:100 into 5 ml MBM medium with 5 mM taurine (Sigma-Aldrich, T0625). Where indicated, 0.5 mM MTHB or DMSHB was added as the substrate, and the samples were incubated at 30 °C for 24 h before the accumulation of DMSHB/DMSP was monitored by GC as above.

### Protein purification

*E.* *coli* BL21 (DE3) cells overexpressing DsyGD, DsyG, DsyD, DSYE and the *G.* *sunshinyii* putative reductase (Supplementary Table 8 and Supplementary Figs. 3 and 9) were collected by centrifugation (20 min, 7,500*g* at 4 °C), washed and resuspended in 25 mM Tris–HCl (pH 8.0), 150 mM NaCl. Overexpressed recombinant proteins were purified by Ni^2+^–NTA (nitrilotriacetic acid) affinity chromatography (GE healthcare), followed by gel filtration on a Superdex200 column (Cytiva), as in Li et al.^80^. The purified proteins were flash frozen in liquid nitrogen and stored at −80 °C until required.

### In vitro MSM, DDC and MR enzyme assays

Where appropriate, recombinant DsyGD, DsyD, DSYE, candidate MR and homologous proteins were assayed for MSM, DDC and MR activity, as in Curson et al.^1^.

For in vitro MSM activity, 0–1,000 μM MTHB, 10–1,000 μM AdoMet and 0.1 μM purified DsyGD/DsyG/DSYE were mixed in a total volume of 100 μl reaction buffer containing 100 mM Tris–HCl (pH 7.0) and incubated at 25 °C for 10 min in triplicate. A total of 15 μl of 20% HCl was added to stop the reactions. The reaction buffers with no enzymes were used as negative controls. MSM activity was determined by detecting *S*-adenosyl-homocysteine (AdoHcy) produced from AdoMet demethylation by HPLC, as described in Li et al.^44^.

For in vitro DDC activity, 0.5–3 mM DMSHB and 0.1 μM purified DsyGD or DsyD domain proteins were mixed in a total volume of 100 μl with reaction buffer (100 mM Tris–HCl (pH 7.0)), before incubation at 25 °C for 10 min in triplicate. A total of 15 μl of 20% HCl was added to stop the reaction. In vitro DDC activity of DsyGD and DsyD was monitored via the HPLC detection of acrylate produced from alkaline hydrolysis of the DMSP reaction product^81,82^.

To determine the optimal temperature of DsyGD and DSYE for MTHB, the reaction mixtures were incubated at 10–60 °C. The optimum pH values of purified enzymes on MTHB were examined at their optimal temperature using Britton–Robinson buffer at pH 4–11, as in Peng et al.^83^.

Kinetic parameters of DsyGD and DSYE for MTHB, AdoMet and DMSHB (for DsyGD) were determined by non-linear analysis based on the initial rates with 0–20,000 μM MTHB, 0–250 μM AdoMet or 500–3,000 μM DMSHB at the optimal temperature and pH, as described in Peng et al.^83^.

For in vitro MR activity, 1 mM MTOB and 0.25 mM NADPH were mixed in a total volume of 2 ml reaction buffer (10 mM Tris–HCl, pH 8.0) in triplicate and incubated at 30 °C. The reactions were initiated by the addition of 1 μM purified reductase enzyme and MR activity was monitored by NADPH reduction at 340 nm using a V550 ultraviolet–visible light spectrophotometre (Jasco) at 0, 15 and 180 min after enzyme addition. The reaction mixtures with no reductase enzyme were used as negative controls.

### Distribution of DMSP synthesis genes in Tara Oceans datasets

To study the relative abundance and distribution of DMSP synthesis genes/transcripts in Tara Oceans OM-RGC_v2 and MATOU datasets^65^, a hidden Markov model profile of reported DMSP synthesis enzymes and experimentally ratified DsyGD, DsyG and DSYE proteins (Supplementary Table 10 and Supplementary Data 1) was created using HMMER tools (v.3.3, http://hmmer.janelia.org/)^84^. The hidden Markov model searches were performed on the online webserver Ocean Gene Atlas^65^ with default settings and an *E*-value of 1 × 10^−30^. The resultant sequences were further verified by BLASTp analysis. Only homologues with ≥40% amino acid identity and ≥70% coverage to ratified sequences were counted. In metagenomic samples, the relative abundance of eukaryotic DMSP synthesis genes was normalized to the relative abundance of *ACTB*, which encodes β-actin, except for *DSYE*, which was also normalized to *recA*. The relative abundance of prokaryotic DMSP synthesis genes was normalized to the relative abundance of *recA*^85^. In metatranscriptomic datasets, the relative abundance of DMSP synthesis transcripts is expressed as percentage of mapped reads. Finally, the biogeographic distribution of DMSP synthesis genes/transcripts was plotted with R (v. 4.0.3) using scatterpie and ggplot2 (ref. ^86^).

### Relative abundance of *dsyGD* in terrestrial metagenomes

The relative abundance of *dsyGD* in metagenomic datasets of *S.* *alterniflora*, *Rhizophora stylosa* and mangrove sediment from the Chinese National Genomics Data Center GSA database (PRJCA002729) was analysed, as in Liu et al.^85^. Only homologues with ≥40% amino acid identity and ≥70% coverage to ratified sequences (Supplementary Table 10 and Supplementary Data 1) were counted.

### Phylogenetic analysis of DMSP synthesis enzymes

All prokaryotic DsyB, MmtN, DsyGD, DsyG, DsyG-like (lacking MSM function) and DsyD sequences, and eukaryotic DSYB, TpMMT and DSYE sequences listed in Supplementary Table 10 were aligned in MAFFT version 7 (ref. ^87^) using default settings, then visually checked. The *S*-methyltransferase or decarboxylase domains sequences of these enzymes were used to construct maximum-likelihood phylogenetic trees using MEGA version X (ref. ^88^) (Fig. 2 and Supplementary Fig. 6). The maximum-likelihood phylogenetic trees were visualized and annotated using the Interactive Tree Of Life version 6.6 (ref. ^89^).

### Statistical methods

All measurements of metabolites, for example, DMSP, DMSHB and DMS levels (in bacterial strains or enzyme assays) were based on the mean of three biological replicates per strain/condition tested, and the error bars indicate standard deviations. For RT–qPCR assays, the results shown represent the mean of three biological replicates and three technical replicates with their respective standard deviations. To identify statistically significant differences between standard and experimental conditions in Figs. 1b and 3a,b, Supplementary Fig. 5a,c and Supplementary Fig. 7a (*P* < 0.05), a two-sided independent Student’s *t*-test was applied to the data. For Supplementary Fig. 11a–d (*P* < 0.05), a Wilcoxon test was applied to the data.

### Reporting summary

Further information on research design is available in the Nature Portfolio Reporting Summary linked to this article.

### Supplementary information

Supplementary Information Supplementary Tables 1, 4 and 7–10 and Figs. 1–12.

Reporting Summary

Supplementary Data 1 Amino acid sequences of proteins tested for functionality in this study.

Supplementary Table 1 Supplementary Tables 2, 3, 5 and 6.

Supplementary Data 5 Source data for Supplementary Figs. 1.

Supplementary Data 5 Source data for Supplementary Figs. 5.

Supplementary Data 5 Source data for Supplementary Figs. 7.

Supplementary Data 5 Source data for Supplementary Figs. 8.

Supplementary Data 5 Source data for Supplementary Figs. 11.

### Source data

Source Data Fig. 1 DMSP production data from three independent biological replicates are displayed in Fig. 1.

Source Data Fig. 3 Data from three independent biological replicates in experiments are shown in Fig. 3.

## Data Availability

Accession numbers of sequences from the NCBI and MMETSP analysed in this study are listed in Table 1 and Supplementary Tables 1 and 10. Source data are provided with this paper.
