# Supplementary material for: Alternative dimethylsulfoniopropionate biosynthesis enzymes in diverse and abundant microorganisms
Source: Nat Microbiol. 2024 Jun 11;9(8):1979–92. doi: 10.1038/s41564-024-01715-9 (PMC11306096; doi:10.1038/s41564-024-01715-9)

nature microbiology

Supplementary information https://doi.org/10.1038/s41564-024-01715-9

Alternative dimethylsulfoniopropionate biosynthesis enzymes in diverse and abundant microorganisms

In the format provided by the authors and unedited

Supplementary information guide to Supplementary Tables 1–10.

Supplementary Table 1:
Homologous proteins to ^{Gs}DsyGD tested in this study.

Supplementary Table 2 (.xlsx):
Proteins from MMETSP homologous to DsyD.

Supplementary Table 3 (.xlsx):
Eukaryotic transcriptomes from the Marine Microbial Eukaryote Transcriptome Sequencing Project (MMETSP) containing DSYE, and if the organisms have been shown to produce DMSP (modified from Li et al., 2022)

Supplementary Table 4:
DMSP levels in DSYE-containing algae assayed by GC.

Supplementary Table 5 (.xlsx):
Average relative abundance of DMSP synthesis methyltransferase genes/transcripts in Tara Oceans datasets. Average relative abundance was calculated by dividing the relative abundance of DMSP synthesis methyltransferases genes/transcripts by the number of Tara Oceans stations in which they were detected.

Supplementary Table 6 (.xlsx):
Relative abundance of algal DMSP synthesis methyltransferase genes in MATOU metagenomes. SRF, surface water layer; DCM, deep chlorophyll maximum layer; MES, mesopelagic zone; MIX, marine epipelagic mixed layer; FSW, filtered sea water; ZZZ, marine water layer.

Supplementary Table 7:

Strains used in this study.

Supplementary Table 8:
Plasmids used in this study.

Supplementary Table 9:
Primers used in this study.

Supplementary Table 10:
Accession numbers of previously ratified enzymes used in bioinformatic analysis.

Supplementary Table 1: Homologous proteins to ^{Gs}DsyGD tested in this study.

Organism and enzyme	Accession Number	Protein length (aa)	Identity to *^{Gs}DsyGD (%)	Coverage to ^{Gs}DsyGD (%)	E-value
G. sunshinyii YC6258 DsyGD	WP044616208.1	494	100	100	0
Symploca sp. SIO3E6 DsyGD	NES17792.1	499	46.04	96	$6e^{-153}$
Oscillatoria sp. SIO1A7 DsyGD	NER39123.1	490	46.60	97	$3e^{-151}$
Zarconia navalis LEGE 11467 DsyG	WP264320056.1	276	49.81	54	$8e^{-85}$
Fragilariopsis cylindrus CCMP1102 DSYE	OEU16654.1	306	38.27	55	$2e^{-56}$
Planctomycetales bacterium DsyG-like	MCA9139260.1	278	38.85	55	$3e^{-60}$
Porphyra umbilicalis DsyGD-like	OSX77567.1	458	38.57	55	$1e^{-55}$
Nitzschia inconspicua DSYE	KAG7362955.1	305	39.43	55	$1e^{-57}$
	**MMETSP ID**	**Protein length (aa)**	**Identity to ※^{Fc}DSYE (%)**	**Coverage to ^{Fc}DSYE (%)**	**E-value**
Norrisiella sphaerica BC52 DSYE	MMETSP0113_16486|m.23051	284	49.65	92	$2e^{-106}$
Pelagococcus subviridis CCMP1429 DSYE	MMETSP0882_30218|m.93541	307	61.46	97	$6e^{-136}$
Bigelowiella longifila CCMP242 DSYE	MMETSP1359_22057|m.30888	304	47.30	95	$1e^{-104}$
Tetraselmis striata LANL1001 DSYE	MMETSP0803_2078|m.7237	340	52.54	96	$7e^{-110}$
Pavlova sp. CCMP459 DSYE	MMETSP1381_18054|m.44509	309	67.79	97	$8e^{-158}$
Ostreococcus prasinos BCC99000 DSYE	MMETSP0933_5616|m.17802	301	56.75	94	$1e^{-114}$
Exanthemachrysis gayraliae RCC1523 DSYE	MMETSP1464_12967|m.43959	338	68.79	97	$9e^{-160}$
Chroomonas mesostigmatica CCMP1168 DSYE	MMETSP0047_26906|m.66305	347	50.87	94	$2e^{-108}$
	**MMETSP ID**	**Protein length (aa)**	**Identity to §^{Os}DsyD (%)**	**Coverage to ^{Os}DsyD (%)**	**E-value**
Prymnesium parvum Texoma1 DsyD-like	MMETSP0008_14016|m.24446	175	33.68	55	$3e^{-12}$
Alexandrium monilatum CCMP3105 DsyD-like	MMETSP0093_31591|m.94061	259	27.78	83	$2e^{-15}$

*^{Gs}DsyGD: aa identity of retrieved sequences to ratified *G. sunshinyii* YC6258 DsyGD.

※^{Fc}DSYE: aa identity of retrieved sequences to ratified *Fragilariopsis cylindrus* CCMP1102 DSYE.

§^{Os}DsyD: aa identity of retrieved sequences to *Oscillatoria* sp. SIO1A7 DsyD.

Supplementary Table 4: DMSP levels in DSYE-containing algae assayed by GC.

Organism	Phylum	Class	DSYE clade	DMSP concentration*
Chrysocystis fragilis RCC 6172	*Ochrophyta*	*Pelagophyceae*	Clade C	86.17 ± 0.28 mM
Aureococcus anophagefferens RCC 4094	*Ochrophyta*	*Pelagophyceae*	Clade C	39.25 ± 2.51 mM
Pelagococcus subviridis RCC 4422	*Ochrophyta*	*Pelagophyceae*	Clade C	51.93 ± 0.90 mM
Chrysoreinhardia sp. RCC 2956	*Ochrophyta*	*Pelagophyceae*	Clade C	233.81 ± 32.10 mM
Pelagomonas calceolata RCC 100	*Ochrophyta*	*Pelagophyceae*	Clade C	13.79 ± 0.46 mM
Ostreococcus tauri	*Chlorophyta*	*Prasinophyceae*	Clade B	0.34 ± 0.003 nmol (μg protein)$^{-1}$

* *Ostreococcus tauri* DMSP production was normalised to protein concentration, while others were normalised to cell volume. Values represent the mean of three independent biological replicates with their respective standard deviations.

Supplementary Table 7: Strains used in this study.

Strain	Description	Reference
Gynuella sunshinyii YC6258	Wild type strain	[1]
Escherichia coli 803	Strain used for routine transformations	[2]
E. coli BL21 (DE3)	Strain for overexpression of cloned genes in pET vectors	[3]
E. coli MC4100	Control strain for osmosensitivity test	Horizon Discovery Biosciences Limited[4]
E. coli FF4169	Strain with *otsA*⁻ mutation, deficient in trehalose production for osmosensitivity tests	Horizon Discovery Biosciences Limited[4]
Rhizobium leguminosarum J391	Streptomycin-resistant derivative of wild type strain 3841 used for library screening	[5]
Labrenzia aggregata dsyB⁻ mutant	*Labrenzia aggregata* LZB033 with a deletion mutation in *dsyB*, used for methyltransferase domain functional assays, rifampicin-resistant.	[6]
Ruegeria pomeroyi DSS-3	Rifampicin-resistant wild type strain used for decarboxylase domain functional assays	[7]
Zarconia navalis LEGE 11467	Cyanobacterium with DsyG	LEGE Culture Collection (LEGE-CC) at CIIMAR, Porto, Portugal[8]
Chrysocystis fragilis RCC 6172	*Pelagophyceae* algae with candidate DSYE	Roscoff Culture Collection (RCC)
Aureococcus anophagefferens RCC 4094	*Pelagophyceae* algae with candidate DSYE	Roscoff Culture Collection (RCC)
Pelagococcus subviridis RCC 4422	*Pelagophyceae* algae with candidate DSYE	Roscoff Culture Collection (RCC)
Chrysoreinhardia sp. RCC 2956	*Pelagophyceae* algae with candidate DSYE	Roscoff Culture Collection (RCC)
Pelagomonas calceolata RCC 100	*Pelagophyceae* algae with candidate DSYE	Roscoff Culture Collection (RCC)

Supplementary Table 8: Plasmids used in this study.

Plasmid	Description	Reference
pLAFR3	Wide host-range cosmid vector, used for library construction, tetracycline-resistant	[9]
pLMB509	Plasmid vector for expression of cloned genes in *L. aggregata dsyB*$^{-}$ mutation and *R. pomeroyi* DSS-3, gentamicin-resistant	[10]
pRK2013	Helper plasmid used in triparental mating, kanamycin-resistant	[11]
pUCm-T	Plasmid vector used for cloning *dsyGD* in *E. coli* FF4169, ampicillin-resistant	Sangon Biotech
pET-16b	Plasmid vector for expression of cloned genes in *E. coli* BL21 (DE3), ampicillin-resistant	Merck Millipore
pET-22b	Plasmid vector for protein purification of cloned genes in *E. coli* BL21 (DE3), ampicillin-resistant	Merck Millipore
pJDT0020	pLAFR3 cosmid from *G. sunshinyii* library that contains ~30 kb genomic DNA including *dsyGD*, tetracycline-resistant	This study
pJDT0021	*G. sunshinyii* YC6258 *dsyGD* cloned in pET-22b	This study
pJDT0022	*G. sunshinyii* YC6258 *dsyG* cloned in pET-22b	This study
pJDT0023	*G. sunshinyii* YC6258 *dsyD* cloned in pET-22b	This study
pJDT0024	*G. sunshinyii* YC6258 reductase domain cloned in pET-22b	This study
pJDT0025	*G. sunshinyii* YC6258 *dsyGD* cloned in pET-16b	This study
pJDT0026	*G. sunshinyii* YC6258 *dsyG* cloned in pET-16b	This study
pJDT0027	*G. sunshinyii* YC6258 *dsyD* cloned in	This study

	pET-16b	
pJDT0028	*G. sunshinyii* YC6258 reductase domain cloned in pET-16b	This study
pJDT0029	*G. sunshinyii* YC6258 *dsyGD* with promoter region cloned in pUCm-T vector	This study
pJDT0030	*Planctomycetales* bacterium *dsyG* cloned in pET-16b	This study
pJDT0031	*Planctomycetales* bacterium *dsyG* cloned in pLMB509	This study
pJDT0032	*P. umbilicalis dsyGD* cloned in pET-16b	This study
pJDT0033	*Z. navalis* LEGE 11467 *dsyG* cloned in pET-16b	This study
pJDT0034	*Z. navalis* LEGE 11467 *dsyG* cloned in pLMB509	This study
pJDT0035	*Oscillatoria* sp. SIO1A7 *dsyGD* cloned in pET-16b	This study
pJDT0036	*Oscillatoria* sp. SIO1A7 *dsyGD* cloned in pLMB509	This study
pJDT0037	*Symploca* sp. SIO3E6 *dsyGD* cloned in pET-16b	This study
pJDT0038	*Symploca* sp. SIO3E6 *dsyGD* cloned in pLMB509	This study
pJDT0039	*Norrisiella sphaerica* BC52 *DSYE* cloned in pET-16b	This study
pJDT0040	*Norrisiella sphaerica* BC52 *DSYE* cloned in pLMB509	This study
pJDT0041	*Pelagococcus subviridis* CCMP1429 *DSYE* cloned in pET-16b	This study
pJDT0042	*Pelagococcus subviridis* CCMP1429 *DSYE* cloned in pLMB509	This study
pJDT0043	*Fragilariopsis cylindrus* CCMP1102 *DSYE* cloned in pET-16b	This study

pJDT0044	*Fragilariopsis cylindrus* CCMP1102 *DSYE* cloned in pLMB509	This study
pJDT0045	*Nitzschia inconspicua DSYE* cloned in pET-16b	This study
pJDT0046	*Nitzschia inconspicua DSYE* cloned in pLMB509	This study
pJDT0047	*Prymnesium parvum* Texoma1 *dsyD* cloned in pET-16b	This study
pJDT0048	*Prymnesium parvum* Texoma1 *dsyD* cloned in pLMB509	This study
pJDT0049	*Alexandrium monilatum* CCMP3105 *dsyD* cloned in pET-16b	This study
pJDT0050	*Alexandrium monilatum* CCMP3105 *dsyD* cloned in pLMB509	This study
pJDT0051	*Bigelowiella longifila* CCMP242 *DSYE* cloned in pET-16b	This study
pJDT0052	*Tetraselmis striata* LANL1001 *DSYE* cloned in pET-16b	This study
pJDT0053	*Pavlova* sp. CCMP459 *DSYE* cloned in pET-16b	This study
pJDT0054	*Ostreococcus prasinos* BCC99000 *DSYE* cloned in pET-16b	This study
pJDT0055	*Exanthemachrysis gayraliae* RCC1523 *DSYE* cloned in pET-16b	This study
pJDT0056	*Chroomonas mesostigmatica* CCMP1168 *DSYE* cloned in pET-16b	This study
pJDT0057	*G. sunshinyii* YC6258 *dsyGD* cloned in pLMB509	This study

Supplementary Table 9: Primers used in this study.

Primer name	Sequence (5' to 3')*	Use
GS_Nde_FOR	GGGAATTCCATATGAAACAAGTCAG	PCR amplification of *dsyGD* from *G. sunshinyii* YC6258
GS_BamH_REV	GCGGATCCTGAGTCCGTCC	PCR amplification of *dsyGD* from *G. sunshinyii* YC6258
MT_Gs_Nde_FoR	GGGAATTCCATATGAAACAGGTTTCTTAC	PCR amplification of methyltransferase domain from *G. sunshinyii* YC6258
MT_Gs_Bam_Rev	CGGGATCCCTAAACGAAAGAACCCCA	PCR amplification of methyltransferase domain from *G. sunshinyii* YC6258
DB_Gs_Nde_FoR	GGGAATTCCATATGAACAAATCTACCCGTC	PCR amplification of decarboxylase domain from *G. sunshinyii* YC6258
DB_Gs_Bam_Rev	CGGGATCCCTAAGAGTCGGTACCGG	PCR amplification of decarboxylase domain from *G. sunshinyii* YC6258
RT_Gs_Nde_FoR	GGGAATTCCATATGAAAAAGATCTT	PCR amplification of reductase domain from *G. sunshinyii* YC6258
RT_Gs_Bam_Rev	CGGGATCCCTAGGAGAACTTTGTGAC	PCR amplification of reductase domain from *G. sunshinyii* YC6258
pLMB_PB_FoR	GGAATTCCATATGCAGAGCGAATT	PCR amplification of *dsyG* from *Planctomycetales* bacterium and cloning into pLMB509
pLMB_PB_Rev	CCGGAATTCGAATGCTCCCC	PCR amplification of *dsyG* from *Planctomycetales* bacterium and cloning into pLMB509
pLMB_LEGE_FoR	GGAATTCCATATGAGCGGCTTCG	PCR amplification of *dsyG* from *Z. navalis* LEGE 11467 and cloning into pLMB509
pLMB_LEGE_Rev	CCGGAATTCACGGGTGTAGAAG	PCR amplification of *dsyG* from *Z. navalis* LEGE 11467 and cloning into pLMB509
pLMB_1A7_FoR	GGAATTCCATATGCTATCTGAAAACCA	PCR amplification of *dsyG* from *Oscillatoria* sp. SIO1A7 and cloning into pLMB509
pLMB_1A7_Rev	CCGGAATTCTTAAAAATTACAGGACTTAC	PCR amplification of *dsyG* from *Oscillatoria* sp. SIO1A7 and cloning

		into pLMB509
pLMB_3E6_FoR	GGGAATTCCATATGCTGCGCA	PCR amplification of *dsyG* from *Symploca* sp. SIO3E6 and cloning into pLMB509
pLMB_3E6_Rev	CCGGAATTCTCAGATGCAAACTTTC	PCR amplification of *dsyG* from *Symploca* sp. SIO3E6 and cloning into pLMB509
pLMB_NS_FoR	GGAATTCCATATGAGCGAAGTTGGTAG	PCR amplification of *DSYE* from *Norrisiella sphaerica* BC52 and cloning into pLMB509
pLMB_NS_Rev	CGGAATTCATTCTGATCATTGACTGC	PCR amplification of *DSYE* from *Norrisiella sphaerica* BC52 and cloning into pLMB509
pLMB_PS_FoR	GGAATTCCATATGGCCGCAAATA	PCR amplification of *DSYE* from *Pelagococcus subviridis* CCMP1429 and cloning into pLMB509
pLMB_PS_Rev	CCGGAATTCATACAGCGGACC	PCR amplification of *DSYE* from *Pelagococcus subviridis* CCMP1429 and cloning into pLMB509
pLMB_FC_FoR	GGAATTCCATATGGCCCCGC	PCR amplification of *DSYE* from *Fragilariopsis cylindrus* CCMP1102 and cloning into pLMB509
pLMB_FC_Rev	CCGGAATTCATAAATATTACCTTCGGT	PCR amplification of *DSYE* from *Fragilariopsis cylindrus* CCMP1102 and cloning into pLMB509
pLMB_NI_FoR	GGAATTCCATATGAGCCCGCTG	PCR amplification of *DSYE* from *Nitzschia inconspicua* and cloning into pLMB509
pLMB_NI_Rev	CCGGAATTCGTAGATATCACCTTCAA	PCR amplification of *DSYE* from *Nitzschia inconspicua* and cloning into pLMB509
pLMB_PP_FoR	GGAATTCCATATGCATGGTGCACA	PCR amplification of *dsyD* from *Prymnesium parvum* Texoma1 and cloning into pLMB509

pLMB_PP_Rev	CCGGAATTCACCACTCGGTTCA	PCR amplification of *dsyD* from *Prymnesium parvum* Texoma1 and cloning into pLMB509
pLMB_AM_FoR	GGAATTCCATATGGCCCGTAGTCG	PCR amplification of *dsyD* from *Alexandrium monilatum* CCMP3105 and cloning into pLMB509
pLMB_AM_Rev	CCGGAATTCACACAGCGGAAC	PCR amplification of *dsyD* from *Alexandrium monilatum* CCMP3105 and cloning into pLMB509
GS_pro_F	ATGAAATCTCTTCAATACGTTTCAGAATCT	PCR amplification of *dsyGD* and promotor from *G. sunshinyii* YC6258 and cloning into pUCm-T vector
GS_pro_R	TGCTGACCGTGATGTTCAACCAC	PCR amplification of *dsyGD* and promotor from *G. sunshinyii* YC6258 and cloning into pUCm-T vector
q_GS_*dsyG*_FOR	AAAATCTCGCCGAACTGGG	RT-qPCR amplification of *dsyG* from *G. sunshinyii* YC6258
q_GS_*dsyG*_REV	CGCTTTGGGATGATCTACC	RT-qPCR amplification of *dsyG* from *G. sunshinyii* YC6258
q_GS_*rpoD*_FOR	TGCTCAACAACCCTGCCTAC	RT-qPCR amplification of *rpoD* from *G. sunshinyii* YC6258
q_GS_*rpoD*_REV	CGATGGAAATGACCAGACGC	RT-qPCR amplification of *rpoD* from *G. sunshinyii* YC6258
q_GS_*recA*_FOR	GGCTCCCCTGAAACCACTA	RT-qPCR amplification of *recA* from *G. sunshinyii* YC6258
q_GS_*recA*_REV	AAAACCTCACCCAAGCGATA	RT-qPCR amplification of *recA* from *G. sunshinyii* YC6258
M13 uni (-43)	AGGGTTTTCCCAGTCACGACGTT	Universal forward primer used to sequence insert ends in pLAFR3
M13 rev (-29)	CAGGAAACAGCTATGACC	Universal reverse primer used to sequence insert ends in pLAFR3

*Restriction sites included in primers are underlined

Supplementary Table 10: Accession numbers of previously ratified enzymes used in bioinformatic analysis.

Protein	Ratified strains	Accession number	Reference
DSYB	*Prymnesium parvum* CCAP946/6	NA	12
	Chrysochromulina tobin CCMP291	KOO32714	
	Lingulodinium polyedrum CCMP1936	NA	
	Alexandrium tamarense ATSP1-B	NA	
	Acropora cervicornis	NA	
	Fragilariopsis cylindrus CCMP1102	OEU17621	
	Symbiodinium microadriaticum CCMP2467	OLQ07620	
TpMMT	*Thalassiosira pseudonana* CCMP1335	Tp23128	13
DsyB	*Labrenzia aggregata* IAM 12614	EAV42226	6
	Pseudooceanicola batsensis HTCC2597	EAQ04968	
	Pelagibaca bermudensis HTCC2601	EAU45958	
	Amorphus coralli DSM 19760	WP_018697905	
MmtN	*Thalassospira profundimaris* PB8B	OAZ15224	14
	Novosphingobium sp. MBES04	GAM03459	
	Roseovarius indicus B108	KRS18724	
	Nocardiopsis chromatogenes YIM 90109	WP_017624909	
	Streptomyces mobaraensis DSM 40847	EME99407	
DmdA	*Ruegeria pomeroyi* DSS-3	AAV95190	15
	Candidatus Pelagibacter ubique HTCC1062	WP_011281570	
	Dinoroseobacter shibae DFL 12	WP_012178987	16
	marine gammaproteobacterium HTCC2080	WP_007233625	
	Candidatus Pelagibacter sp. HTCC7211	WP_008546106	17
	Candidatus Puniceispirillum marinum IMCC1322	WP_013044947	
DddD	*Marinomonas* sp. MWYL1	ABR72937	18
	Oceanimonas doudoroffii	AEQ39135	19
	Psychrobacter sp. J466	ACY02894	
	Halomonas sp. HTNK1	ACV84065	20
	Sinorhizobium fredii NGR234	AAQ87407	18
	Burkholderia ambifaria AMMD	WP_011659284	
	Pseudomonas sp. J465	ACY01992	21
DddL	*Sulfitobacter* sp. EE-36	ADK55772	22
	Rhodobacter sphaeroides 2.4.1	YP_351475	
	Labrenzia aggregata LZB033	KP639184	6
	Ahrensia marina LZD062	KP639183	23
DddP	*Roseovarius nubinhibens* ISM	EAP77700	24
	Ruegeria pomeroyi DSS-3	WP_044029245	7
	Phaeobacter inhibens DSM 17395	AFO91571	25
	Oceanimonas doudoroffii DSM 7028	AEQ39091	19
	Oceanimonas doudoroffii DSM 7028	AEQ39103	
	Aspergillus oryzae RIB40	BAE62778	24

	Fusarium graminearum PH-1	XP_389272	
DddQ	*Ruegeria pomeroyi* DSS-3	WP_011047333	7
	Roseovarius nubinhibens ISM	EAP76002	
	Roseovarius nubinhibens ISM	EAP76001	
	Ruegeria lacuscaerulensis ITI1157	WP_005978225	26
	GOS_2632696	ECW91654	7
	GOS_7860946	EBP74803	
	GOS_2469775	ECX82089	
DddW	*Ruegeria pomeroyi* DSS-3	AAV93771	27
DddY	*Alcaligenes faecalis* M3A	ADT64689	28
	Desulfovibrio acrylicus	SHJ73420	29
	Acinetobacter bereziniae	ENV21217	30
	Ferrimonas kyonanensis DSM 18153	WP_028114584	31
	Shewanella putrefaciens CN-32	ABP77243	32
DddK	*Candidatus* Pelagibacter ubique HTCC1062	AAZ21215	33
	Candidatus Pelagibacter ubique HTCC9022	WP_028037226	
	alphaproteobacterium_HIMB5	AFS47241.1	
DddX	*Marinobacterium jannaschii*	WP_084332639.1	34
	Pelagicola sp. LXJ1103	WP_109384856.1	
	Psychrobacter sp. P11G5	WP_068035783.1	
	Sporosarcina sp. P33	WP_081242855.1	
Alma1	*Emiliania huxleyi* CCMP1516	XP_005784450	35
	Emiliania huxleyi CCMP1516	XP_005763983	
MddA	*Mycobacterium tuberculosis* H37Rv	WP_003416945.1	36
	Bradyrhizobium diazoefficiens USDA 110	WP_011084036.1	
	Bradyrhizobium diazoefficiens USDA 110	WP_011088485.1	
	Pseudomonas sp. GM41	WP_008148420.1	
	Pseudomonas deceptionensis	WP_048359798.1	
	Sulfurovum sp. NBC37-1	WP_011980608.1	
DmdB	*Ruegeria pomeroyi*	WP_011047771.1	37
	Ruegeria pomeroyi	WP_011046428.1	
	Candidatus Pelagibacter ubique	WP_011281571.1	
DmdC	*Ruegeria pomeroyi*	WP_011049476.1	38
	Burkholderia thailandensis E264	WP_009892931.1	
	Ruegeria pomeroyi DSS-3	WP_011048615.1	
	Pseudomonas aeruginosa PAO1	WP_003114720.1	
	Pseudomonas aeruginosa PAO1	WP_003114561.1	
	Burkholderia thailandensis E264	WP_009889880.1	
	Ruegeria lacuscaerulensis ITI-1157	EEX08676.1	
DmdD	*Ruegeria pomeroyi*	Q5LLW6.1	37,38

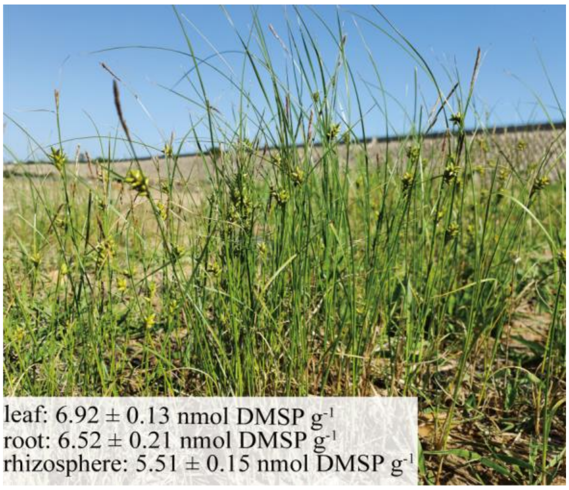

Supplementary Figure 1: DMSP levels in *Carex scabrifolia* plants and associated soil. *C. scabrifolia* plants were sampled in biological triplicates near Qingdao, Shandong Province, China (120.745°E, 36.454°N). DMSP concentrations in *C. scabrifolia* leaves, roots and rhizosphere soil were measured by GC and normalised to fresh weight of plant tissue / soil. Values shown represent the mean of triplicate biological samples with their respective standard deviations.

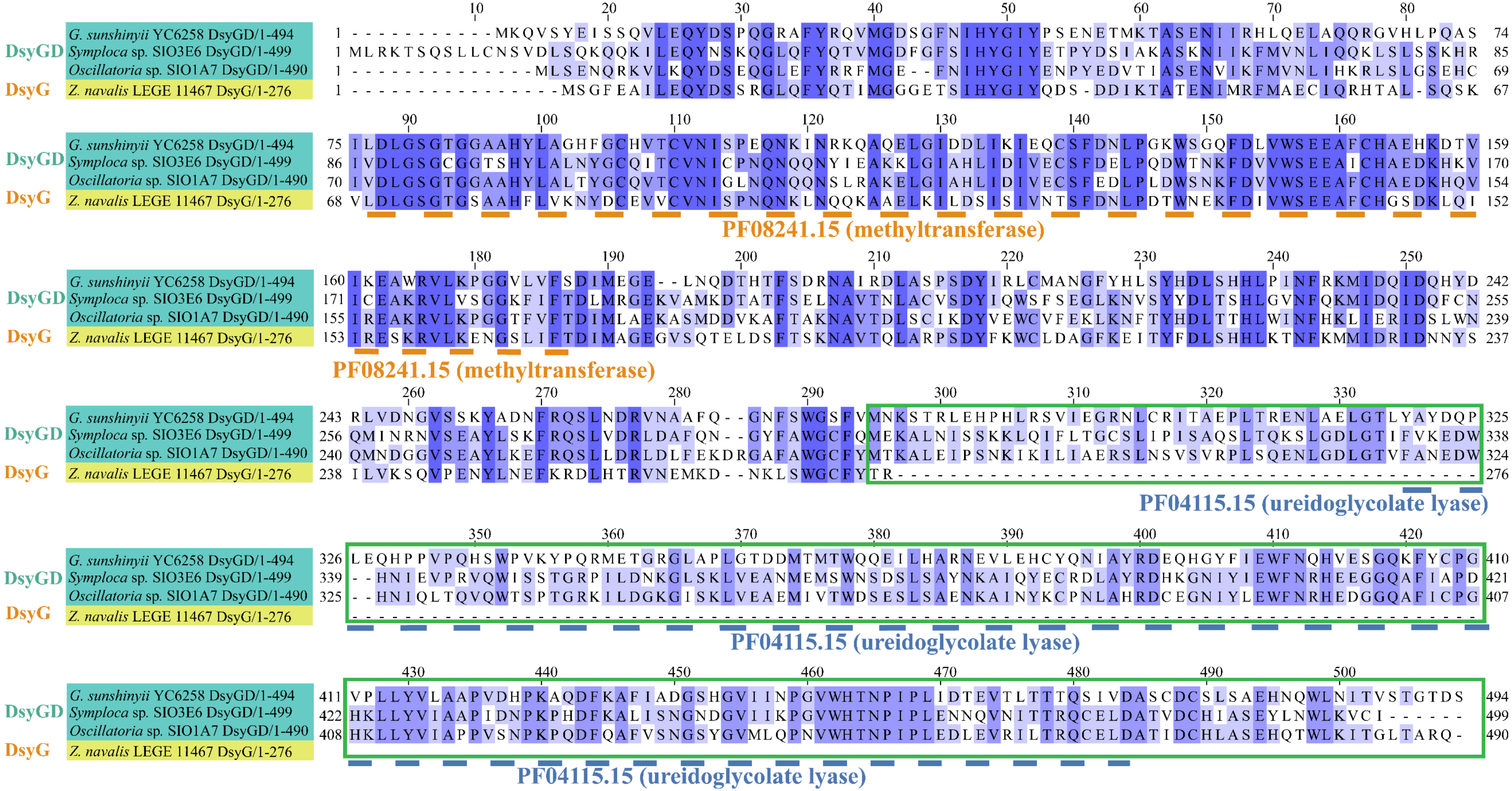
DsyGD
DsyG
G. sunshinyii YC6258 DsyGD/1-494
Symploca sp. SIO3E6 DsyGD/1-499
Oscillatoria sp. SIO1A7 DsyGD/1-490
Z. navalis LEGE 11467 DsyG/1-276
PF08241.15 (methyltransferase)
PF04115.15 (ureidoglycolate lyase)

Supplementary Figure 2: Alignment of DsyGD and DsyG polypeptides from DMSP-producing bacteria. Residues marked in dark, medium and light blue are, respectively, 100%, > 80% and > 60% conserved in the sequences. Residues 76-175 and 320-469 of ^{Gs}DsyGD, indicated by dashed lines, are homologous to the PF08241.15 (methyltransferase) and PF04115.15 (ureidoglycolate lyase) domains, respectively. The green box denotes the truncated ^{Gs}DsyD domain sequence (from the methionine as position 1) that was used to analyse its activity. The N-terminal region immediately upstream of the boxed sequence was used to analyse the activity of the ^{Gs}DsyG domain. Turquoise shading indicates bacteria with double domain DsyGD, whereas light green indicates a single domain DsyG.

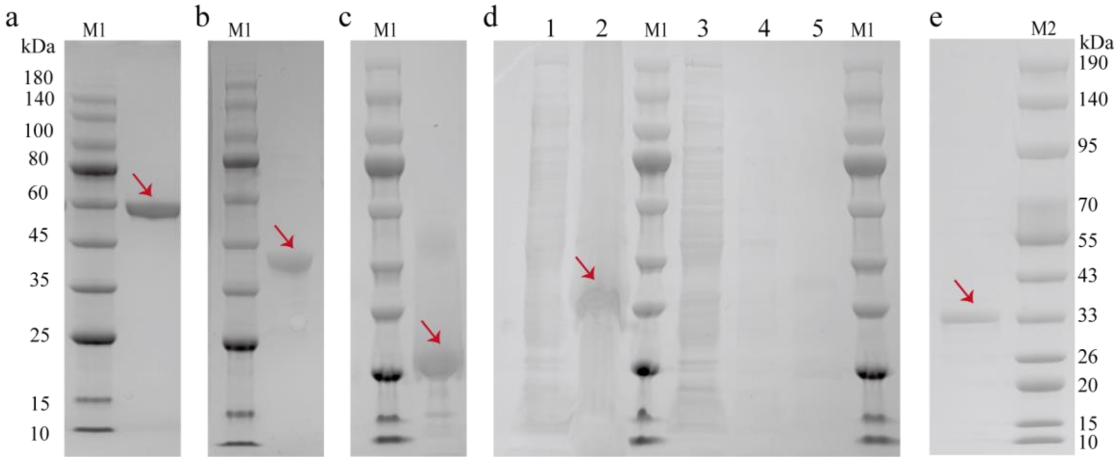

Supplementary Figure 3: SDS-PAGE analysis of ^{Gs}DsyGD, ^{Gs}DsyG, ^{Gs}DsyD, ^{Zn}DsyG and the *Gynuella sunshinyii* putative reductase proteins. Purified recombinant His-tagged proteins were run on 12% precast SDS-PAGE gels. **(a)** ^{Gs}DsyGD (56.0 kDa). **(b)** *G. sunshinyii* candidate reductase protein (35.8 kDa). **(c)** truncated ^{Gs}DsyD (24.4 kDa). **(d)** Insoluble truncated ^{Gs}DsyG (31.7 kDa), 1, soluble lysate before purification; 2, insoluble material in cell lysate; 3, eluate after loading cell lysate; 4, eluate of washing buffer; 5, eluted fraction with imidazole. **(e)** ^{Zn}DsyG (31.6 kDa). M1, Prestained Protein Ladder, 10 to 180 kDa (TransGen); M2, Prestained Protein Ladder, 10 to 190 kDa (TransGen). All protein purifications and SDS-PAGE gels were done twice and representative images are presented.

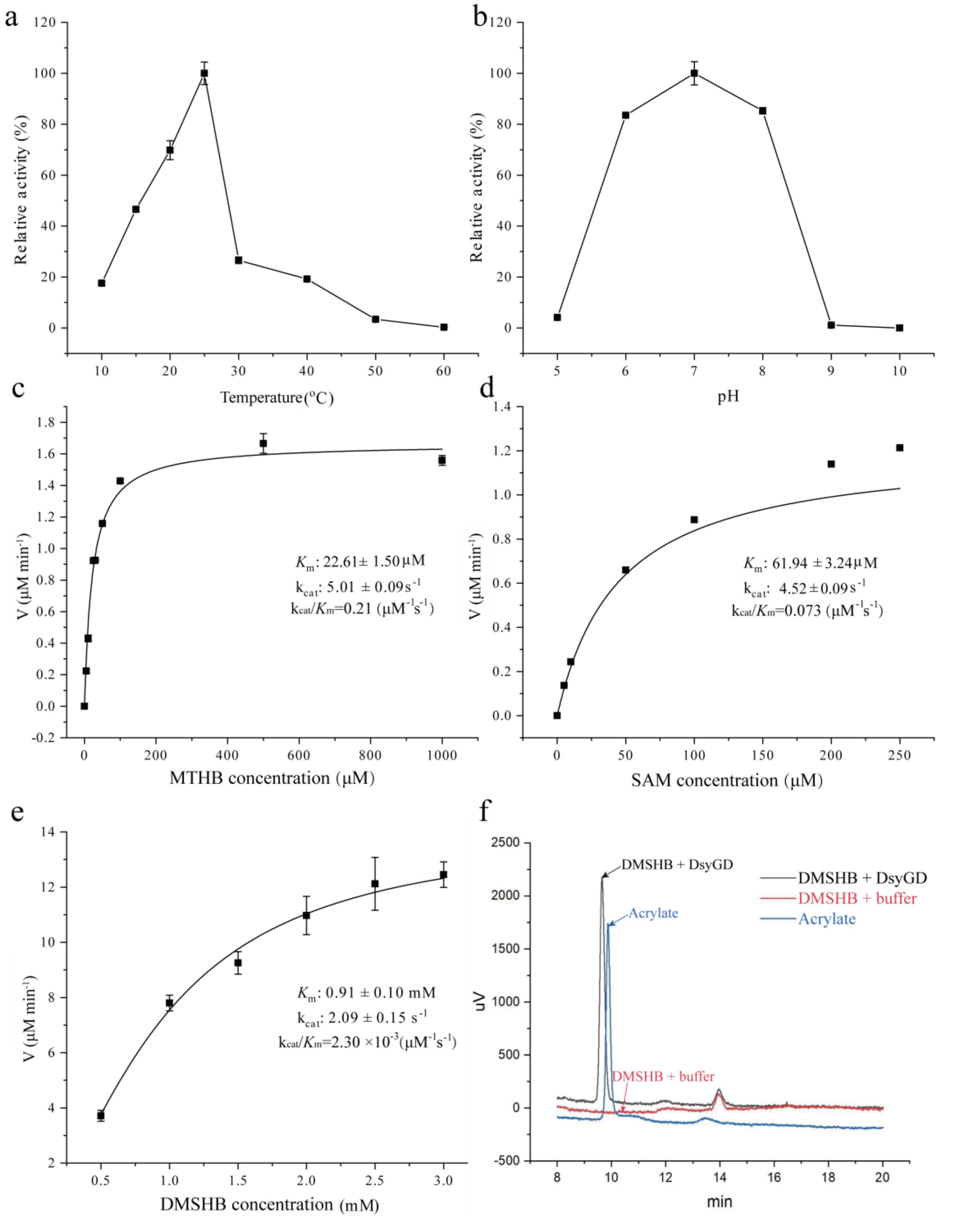

Supplementary Figure 4: Characterisation of recombinant ^{Gs}DsyGD activity by HPLC. (a) Effect of temperature on ^{Gs}DsyGD MSM activity. **(b)** Effect of pH on ^{Gs}DsyGD MSM activity. **(c)** Non-linear fit curve of ^{Gs}DsyGD MSM activity with varying MTHB concentrations. **(d)** Non-linear fit curve of ^{Gs}DsyGD activity with varying SAM concentrations. MSM activity was measured by monitoring *S*-adenosyl homocysteine (SAH) production from demethylation of SAM in both c and d. **(e)** Non-linear fit curve of ^{Gs}DsyGD DDC activity with varying DMSHB concentrations. The kinetic parameters were determined at pH 7.0 and 25°C. **(f)** Detection of acrylate produced by alkaline lysis of DMSP formed from ^{Gs}DsyGD-dependent decarboxylation of DMSHB (black line). The acrylate standard is indicated by a blue line. Incubation of

DMSHB in buffer controls yielded no acrylate (red line). For panels a-e, results shown represent the mean of three independent biological replicates with error bars showing the respective standard deviations (unless they are too small to visualise). Panel f shows single HPLC traces overlaid for three different samples.

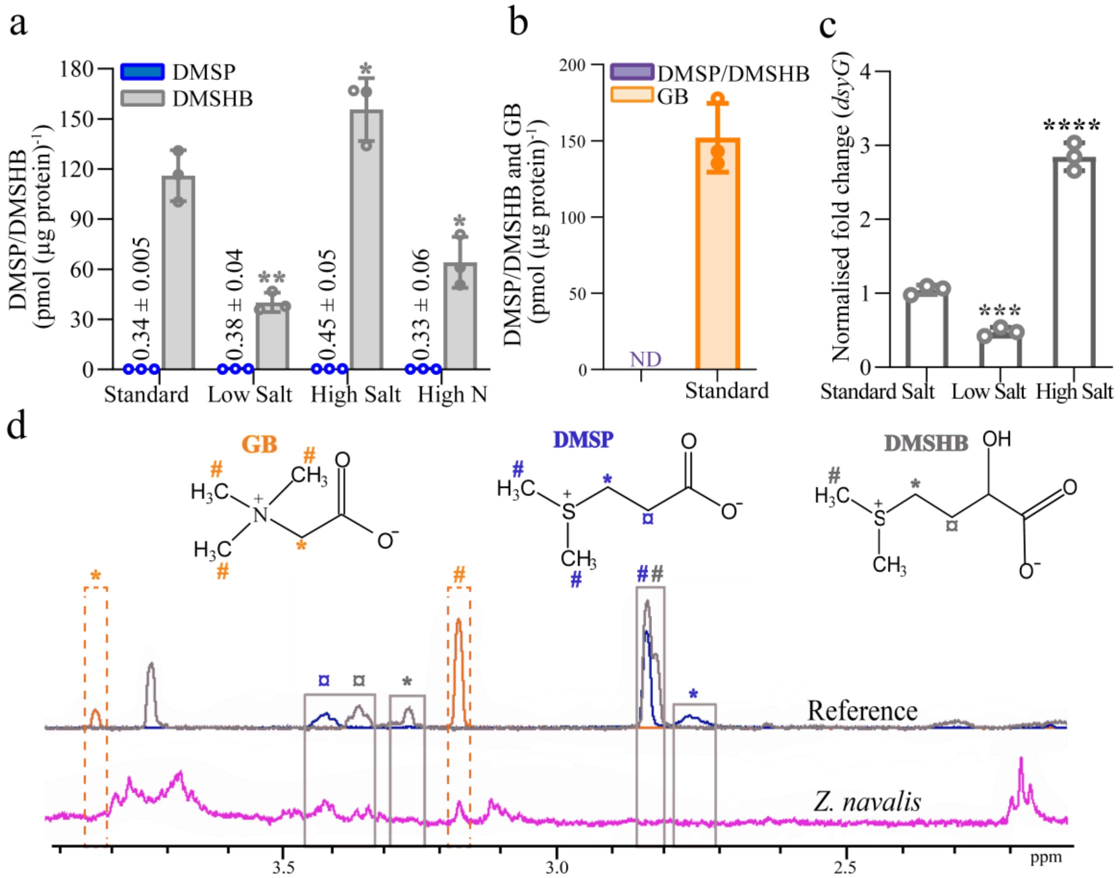

Supplementary Figure 5: DMSP/DMSHB and glycine betaine (GB) in *Zarconia navalis* LEGE 11467. (a) DMSP and DMSHB detected by GC under different stress conditions in *Z. navalis*. Standard condition: 25 PSU (practical salinity units) and 0.5 mM $NaNO_3$; Low salt: 5 PSU with 0.5 mM $NaNO_3$; High salt: 50 PSU with 0.5 mM $NaNO_3$; High nitrogen: 25 PSU with 17.65 mM $NaNO_3$. DMSP levels are indicated above the corresponding columns. **(b)** NMR estimation of DMSP/DMSHB and GB production in *Z. navalis* samples grown under standard conditions. Note, DMSP/DMSHB levels were below the detection limit. ND, not detected. **(c)** Normalised fold change of ^{Zn}*dysG* transcription from *Z. navalis* cultures grown under different salinities (all with 17.65 mM $NaNO_3$). Gene expression was normalised to *recA* and *GADPH*. **(d)** An example NMR spectrum of *Z. navalis* LEGE 11467 cultured in standard conditions, and the spectra for standards of GB (orange spectrum, in orange dashed line) and DMSP/DMSHB (blue and grey spectrum, respectively). DMSP and DMSHB signals (framed in grey continuous line) were overlaid. Experiments in panels a and b show mean values for three independent biological replicates. For RT-qPCR assays in panel c, mean values are shown for three technical replicates for each of three biological replicates. Error bars in panels a-c indicate standard deviation. Statistical significance in panels a and c was determined by two-sided student's *t*-tests (panel a: Low salt: $**p = 0.0013$; High salt: $*p = 0.047$; High N: $**p = 0.0141$; panel c: Low salt: $***p = 0.0004$; High salt: $****p = 0.00009778$).

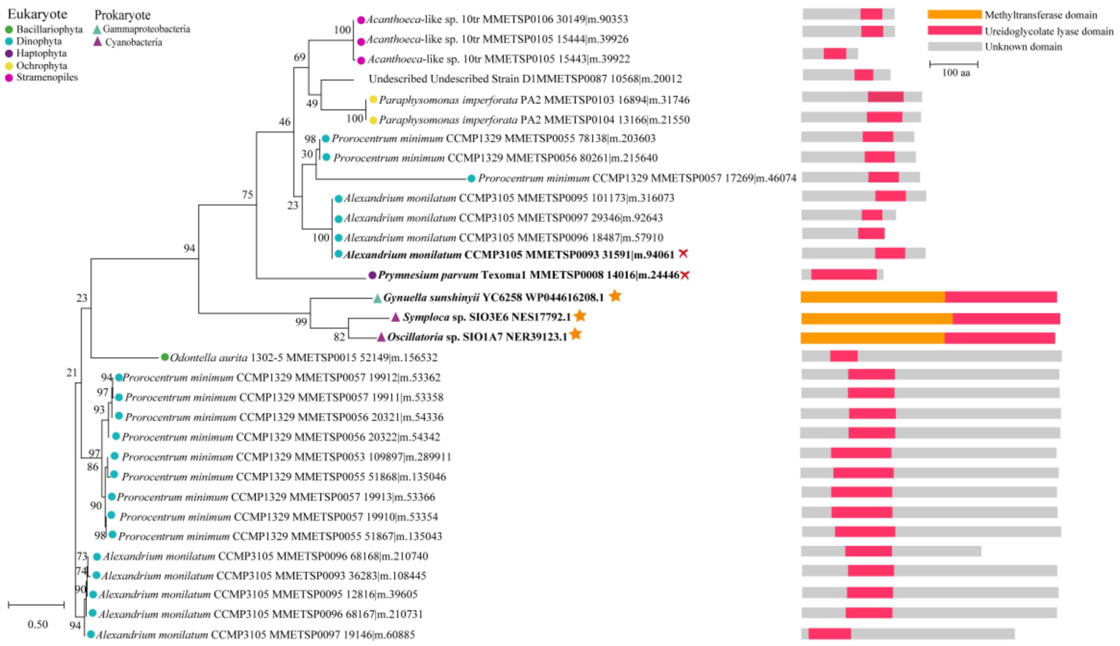

Supplementary Figure 6: Maximum likelihood phylogenetic tree of proteins containing a DsyD-like domain from the MMETSP algal transcriptome database. The tree was constructed in MEGA X with proteins obtained from an HMM search using functional DsyD domains from the *G. sunshinyii* YC6258, *Symploca* sp. SIO3E6 and *Oscillatoria* sp. SIO1A7 DsyGD proteins (labelled with an orange star). DsyD proteins shown to be non-functional are labelled with a red cross. Bootstrap support values are shown. Predicted protein domains are indicated on the right panel and described in the key. Note, DsyD contains a ureidoglycolate lyase domain. Domain analysis was performed by Pfam online server.

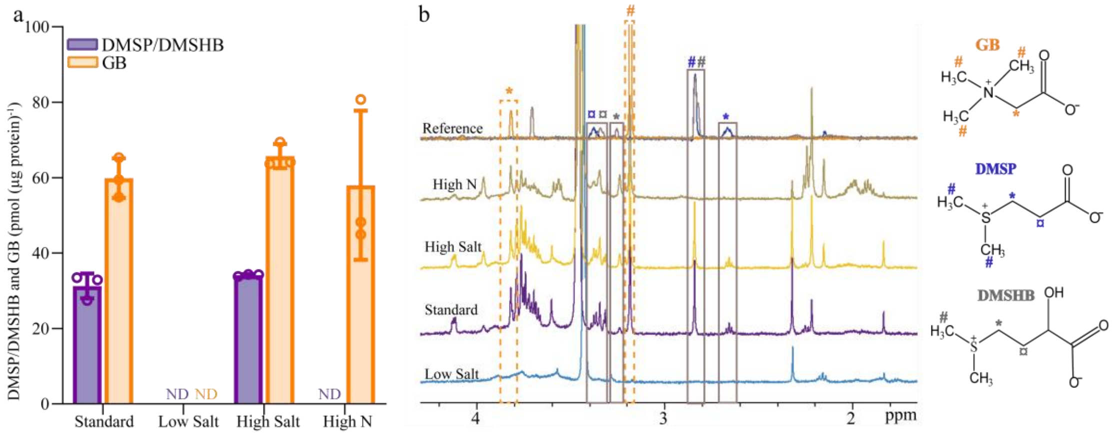

Supplementary Figure 7: DMSP/DMSHB and glycine betaine (GB) in *G. sunshinyii* YC6258 (a) Estimated GB and DMSHB/DMSP production quantified by NMR. A two-sided student's *t*-test was used to assess statistically significant differences between standard conditions (35 PSU (practical salinity units), 0.5 mM NH_4Cl) and different stress conditions. Low salt: 5 PSU, High salt: 50 PSU, High N: 10 mM NH_4Cl. Mean values for three independent biological replicates are shown. Error bars indicate standard deviations. ND, not detected. **(b)** NMR spectra of *G. sunshinyii* grown in the 4 different culture conditions, with the standard spectra for GB (orange spectrum, signals were framed in orange dashed line) and DMSP/DMSHB (blue and grey spectrum, respectively). DMSP and DMSHB signals (framed in grey continuous line) were overlaid. Single replicates are shown in the figure for clarity.

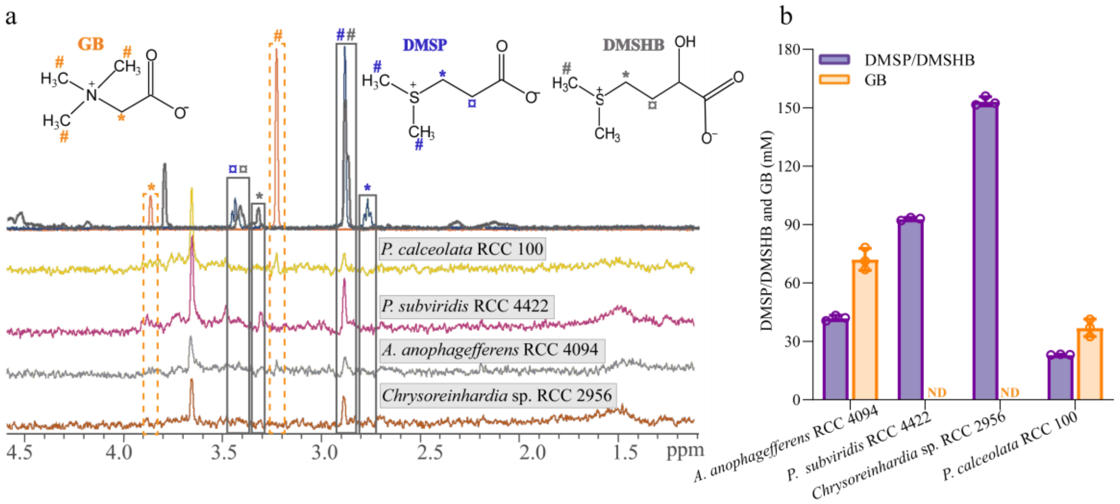

Supplementary Figure 8: DMSP/DMSHB and GB in pelagophyte algae. (a) NMR spectra of pelagophyte algae. The assignment is shown for both GB and DMSP/DMSHB. All the methyl (CH_3) and methylene (CH_2) groups were visible in the samples, but only methyl groups were used for estimation as they were larger and more isolated from each other. Single replicates are shown in the figure for clarity. The reference spectrum is the standard spectra for GB (orange spectrum, in orange dashed line) and DMSP/DMSHB (blue and grey spectrum, respectively). DMSP and DMSHB signals (framed in grey continuous line) were overlaid. **(b)** Estimated cellular DMSP/DMSHB and GB concentrations based on NMR analysis and normalised to cell volume. GB was only detected in *Aureococcus anophagefferens* RCC 4094 and *Pelagomonas calceolata* RCC 100. DMSP/DMSHB was detected in all pelagophytes examined by NMR. Mean values are shown for three biologically independent samples. Error bars indicate standard deviations. ND, not detected.

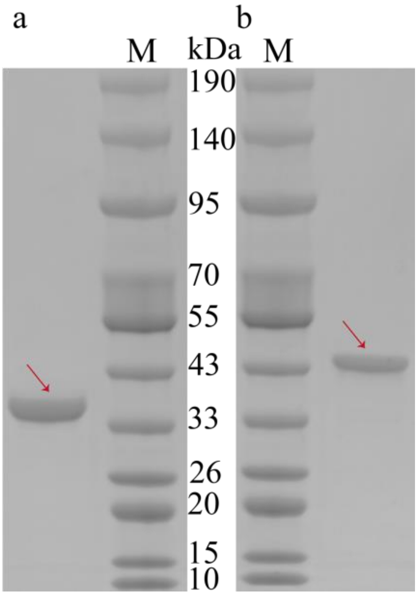

Supplementary Figure 9: Purified DSYE from *Ostreococcus prasinos* BCC99000 and *Chroomonas mesostigmatica* CCMP1168. The purified recombinant His-tagged **(a)** Clade B DSYE (34.0 kDa) from *O. prasinos* BCC99000. **(b)** Clade C DSYE (38.0 kDa) from *C. mesostigmatica* CCMP1168 run on 12% precast SDS-PAGE gels. M, Prestained Protein Ladder, 10 to 190 kDa (TransGen). All protein purifications and SDS-PAGE gels were done twice and representative images are presented.

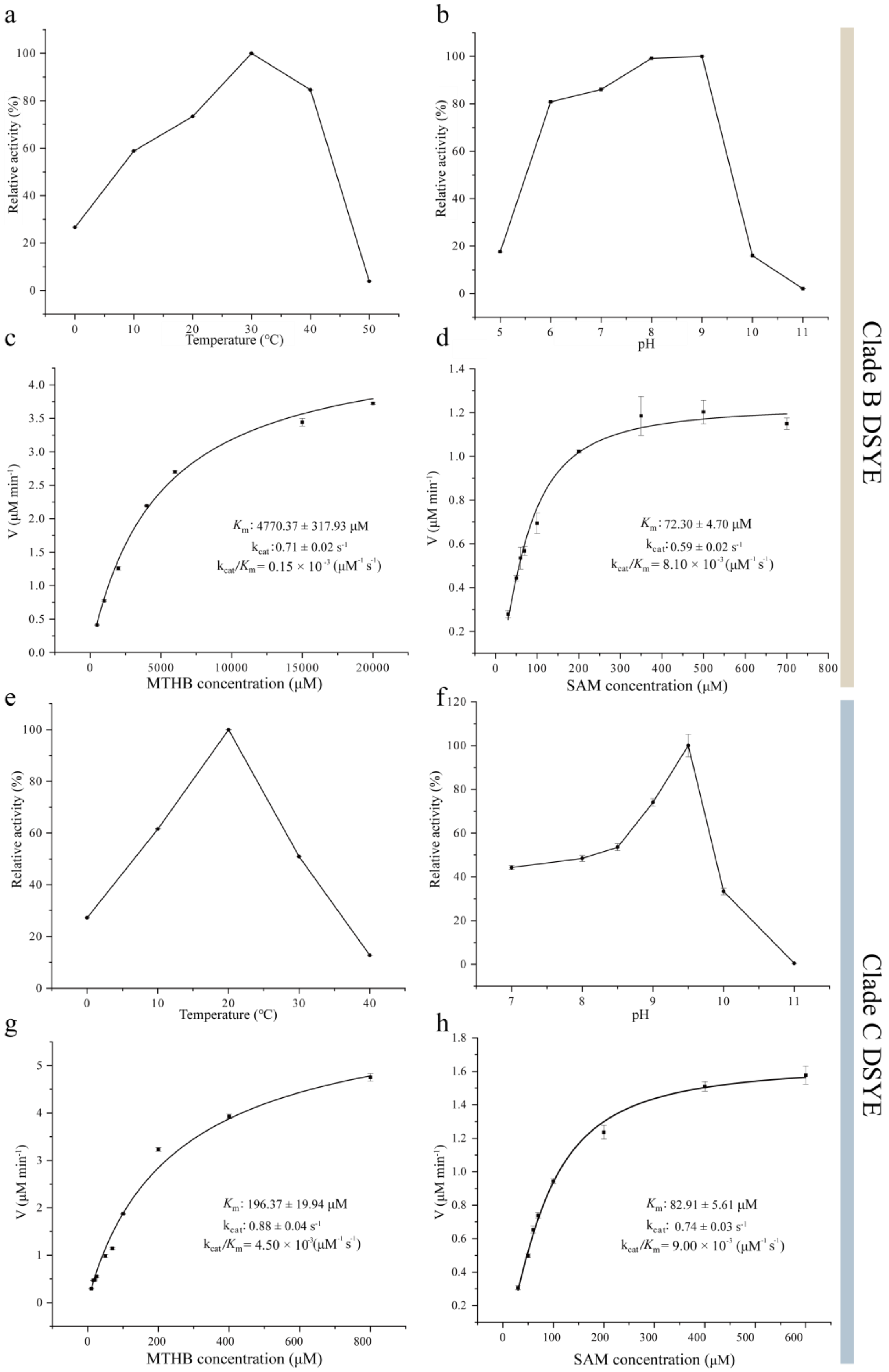

Supplementary Figure 10: Characterisation of recombinant Clade B and Clade C DSYE activity by HPLC. Protein characterisation of Clade B DSYE from *O. prasinos* BCC99000 **(a-d)** and Clade C DSYE from *C. mesostigmatica* CCMP1168 **(e-h)**. **(a)**

and **(e),** Effect of temperature on DSYE MSM activity. **(b)** and **(f),** Effect of pH on DSYE MSM activity. **(c)** and **(g),** Non-linear fit curves of DSYE MSM activity with varying MTHB concentrations. **(d)** and **(h),** Non-linear fit curves of DSYE activity with varying SAM concentrations. In panels c,d, and g-h, MSM activity was measured by monitoring the production of *S*-adenosyl homocysteine (SAH) produced by the demethylation of SAM. All data represent mean values of three independent biological replicates with error bars indicating standard deviation.

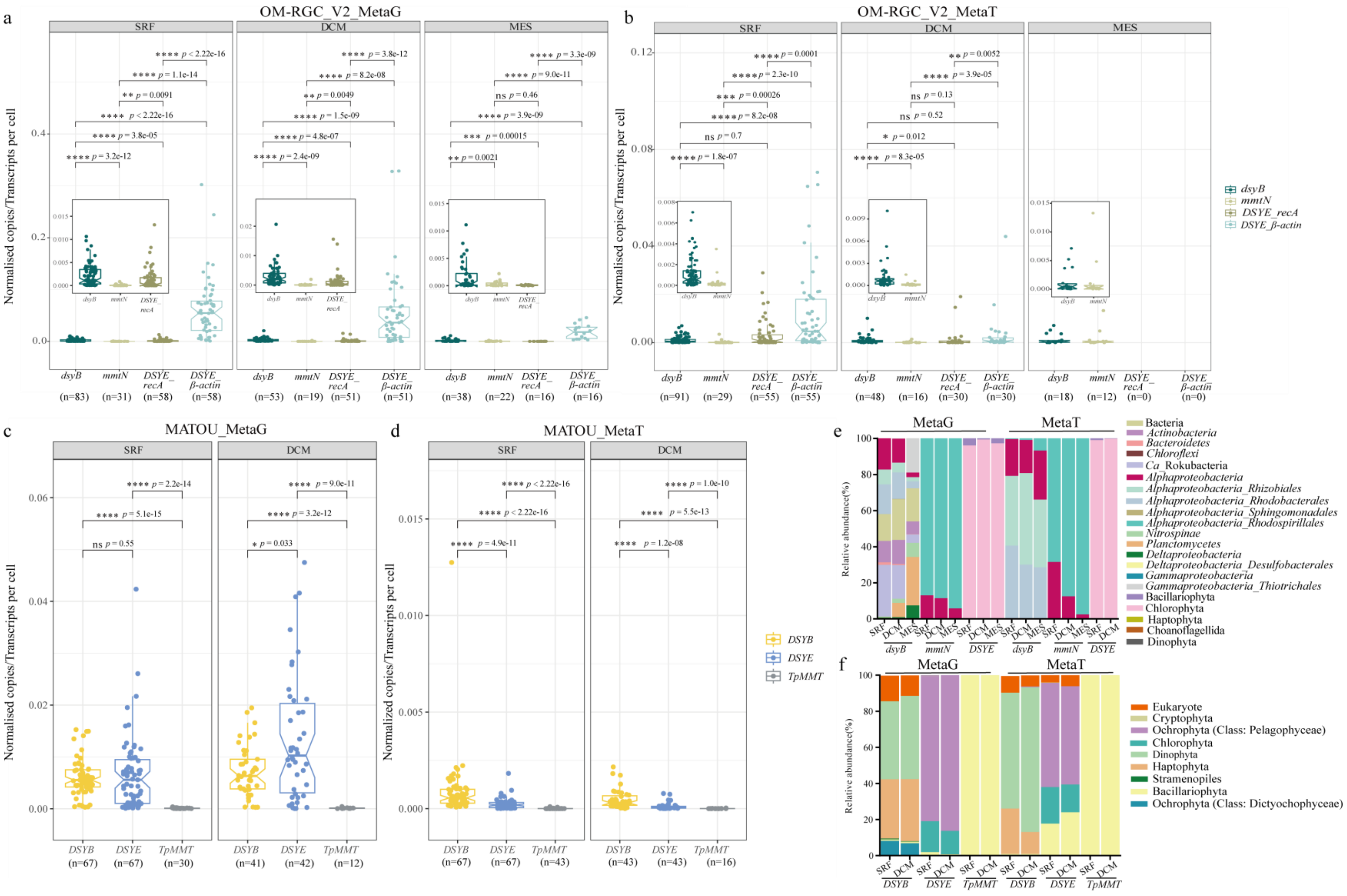
a
OM-RGC_V2_MetaG
SRF
DCM
MES
Normalised copies/Transcripts per cell
dsyB
mmtN
DSYE_ recA
DSYE_ β-actin
(n=83) (n=31) (n=58) (n=58)
(n=53) (n=19) (n=51) (n=51)
(n=38) (n=22) (n=16) (n=16)
b
OM-RGC_V2_MetaT
(n=91) (n=29) (n=55) (n=55)
(n=48) (n=16) (n=30) (n=30)
(n=18) (n=12) (n=0) (n=0)
dsyB
mmtN
DSYE_recA
DSYE_β-actin
c
MATOU_MetaG
SRF
DCM
DSYB
DSYE
TpMMT
(n=67) (n=67) (n=30)
(n=41) (n=42) (n=12)
d
MATOU_MetaT
Normalized copies/Transcripts per cell
(n=67) (n=67) (n=43)
(n=43) (n=43) (n=16)
e
MetaG
MetaT
Relative abundance(%)
SRF DCM MES
Bacteria
Actinobacteria
Bacteroidetes
Chloroflexi
Ca_Rokubacteria
Alphaproteobacteria
Alphaproteobacteria_Rhizobiales
Alphaproteobacteria_Rhodobacterales
Alphaproteobacteria_Sphingomonadales
Alphaproteobacteria_Rhodospirillales
Nitrospinae
Planctomycetes
Deltaproteobacteria
Deltaproteobacteria_Desulfobacterales
Gammaproteobacteria
Gammaproteobacteria_Thiotrichales
Bacillariophyta
Chlorophyta
Haptophyta
Choanoflagellida
Dinophyta
f
MetaG
MetaT
Relative abundance(%)
Eukaryote
Cryptophyta
Ochrophyta (Class: Pelagophyceae)
Chlorophyta
Dinophyta
Haptophyta
Stramenopiles
Bacillariophyta
Ochrophyta (Class: Dictyochophyceae)

Supplementary Figure 11: Distribution of DMSP synthesis genes and transcripts in Tara Oceans datasets. (a, b) Distribution of the DMSP synthesis *S*-methyltransferase genes *dsyB, dsyGD/dsyG*, *DSYE* and *mmtN* in the OM-RGC_V2 metagenomes and metatranscriptomes apportioned to bacterioplankton. No *DSYE* sequences were detected in the MES layer of OM-RGC_V2 metatranscriptomes. **(c, d)**, Distribution of eukaryotic DMSP synthesis *S*-methyltransferase genes *DSYB, DSYE* and *TpMMT* in MATOU metagenomes and metatranscriptomes. All data were divided into surface water layer (SRF) and deep chlorophyll maximum layer (DCM) for the MATOU dataset and the mesopelagic water layer (MES) for the OM-RGC_V2 dataset. Statistically significant differences between water layers in panels a-d were determined by a two-sided Wilcoxon test (*p* values are indicated on the graph; ns, not statistically significant differences). The number of stations in which each DMSP synthesis *S*-methyltransferase genes / transcripts were detected (n) is indicated in the figure. MetaG, metagenomes data; MetaT, metatranscriptomes data. All prokaryotic genes were normalised to *recA*. *DSYE* from OM-RGC_V2 dataset was normalised to both *recA* and *β-actin*. All eukaryotic genes from the MATOU dataset were normalised to *β-actin*. Boxplots show median (centre line), upper and lower quartiles (box limits), the interquartile range (whiskers). Points represent individual data values. **(e)** Taxonomic affiliation of *dsyB*, *DSYE* and *mmtN* sequences in OM-RGC_V2 datasets; **(f)** Taxonomic assignment of *DSYB*, *DSYE* and *TpMMT* sequences in MATOU datasets. Note, no *dsyG*/*dsyGD* sequences were detected in OM-RGC_V2 datasets.

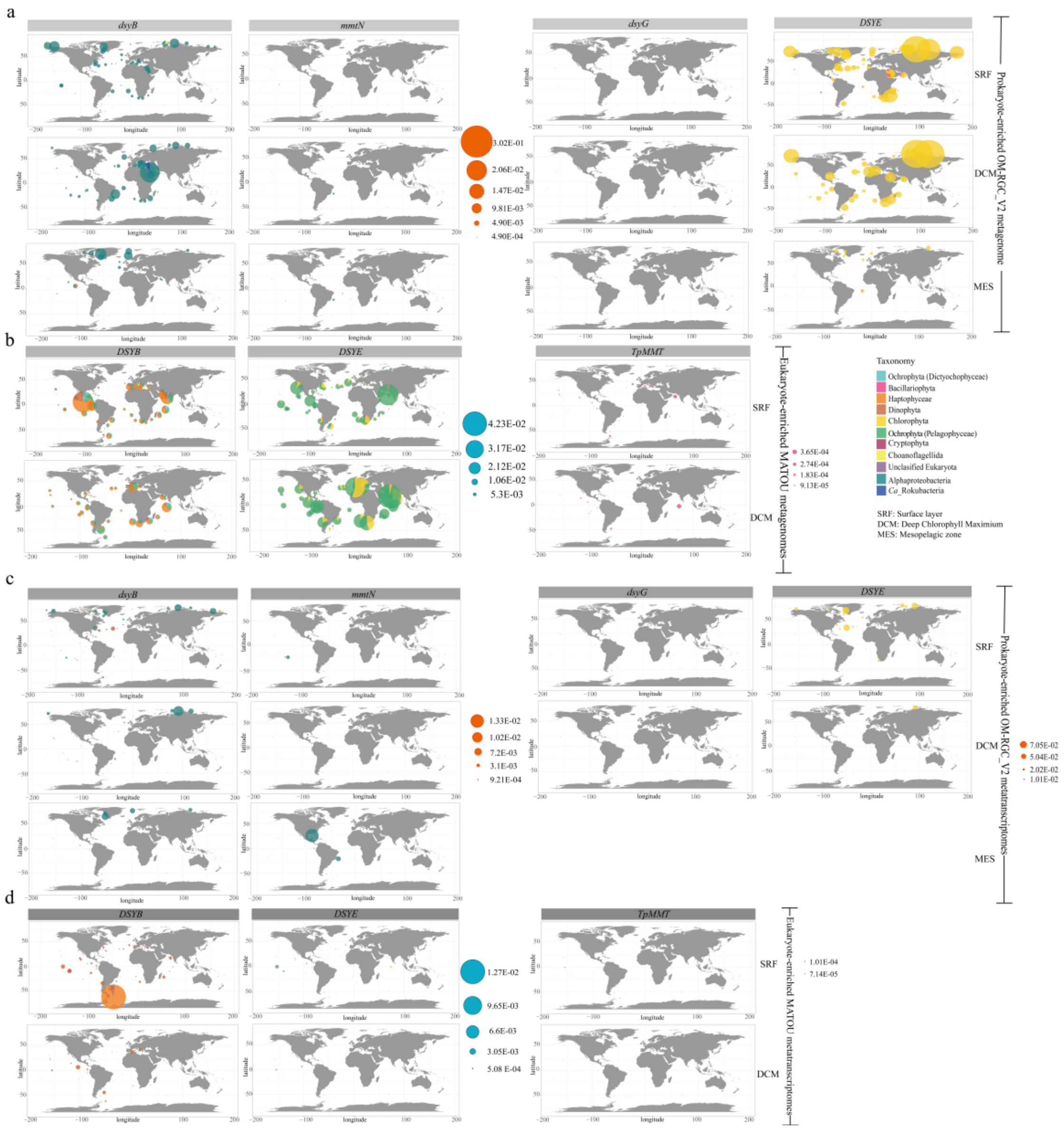

Supplementary Figure 12: Biogeographic distribution and taxonomic composition of methyltransferase genes involved in DMSP synthesis in Tara Ocean datasets. (a) Methyltransferase genes in the prokaryote-enriched OM-RGC_V2 metagenomes. No *dsyG* domain sequences were detected. **(b)** Methyltransferase genes in phytoplankton-enriched MATOU metagenomes. **(c)** Methyltransferase transcripts in the prokaryote-enriched OM-RGC_V2 metatranscriptomes. No *dsyG* domain transcripts were detected in OM-RGC_V2 datasets. **(d)** Methyltransferase transcripts in the eukaryote-enriched MATOU metatranscriptomes. SRF, surface water layer (SRF); deep chlorophyll maximum layer (DCM); MES, mesopelagic water layer (MES).

Supplementary References

1. Chung, E. J., Park J. A., Jeon, C. O. & Chung, Y. R. *Gynuella sunshinyii* gen. nov., sp. nov., an antifungal rhizobacterium isolated from a halophyte, *Carex scabrifolia* Steud. *Int. J. Syst. Evol. Microbiol.* **65,** 1038-1043 (2015).

2 Wood, W. B. Host specificity of DNA produced by *Escherichia coli*: bacterial mutations affecting the restriction and modification. *J. Mol. Biol.* **16,** 118-133 (1966).

3. Studier, F. W. & Moffatt, B. A. Use of bacteriophage T7 RNA polymerase to direct selective high-level expression of cloned genes. *J. MoZ. Biol.* **189,** 113-130 (1986).

4. Giaever, H. M., Styrvold, O. B., Kassen, I. & Strøm, A. R. Biochemical and genetic characterization of osmoregulatory trehalose synthesis in *Escherichia coli. J. Bacteriol.* **170,** 2841-2849 (1988).

5. Young, J. P. W. et al. The genome of *Rhizobium leguminosarum* has recognizable core and accessory components. *Genome Biol.* **7,** R34 (2006).

6. Curson, A. R. J. et al. Dimethylsulfoniopropionate biosynthesis in marine bacteria and identification of the key gene in this process. *Nat. Microbiol.* **2,** 17009 (2017).

7. Todd, J. D. et al. DddQ, a novel, cupin-containing, dimethylsulfoniopropionate lyase in marine roseobacters and in uncultured marine bacteria. *Environ. Microbiol.* **13,** 427-438 (2011).

8. Hentschke, G. S., et al. *Zarconia navalis* gen. nov., sp. nov., *Romeriopsis navalis* gen. nov., sp. nov. and *Romeriopsis marina* sp. nov., isolated from inter- and subtidal environments from northern Portugal. *Int. J. Syst. Evol. Microbiol*. **72**, 005552 (2022).

9. Staskawicz, B., Dahlbeck, D., Keen, N. & Napoli, C. Molecular characterization of cloned Avirulence genes from Race 0 and Race 1 of *Pseudomonas syringae* pv. *glycinea*. *J. Bacteriol.* **169,** 5789-5794 (1987).

10. Tett, A. J., Rudder, S. J., Bourdes, A., Karunakaran, R. & Poole, P. S. Regulatable vectors for environmental gene expression in *Alphaproteobacteria*. *Appl. Environ. Microb*. **78**, 7137–7140 (2012).

11. Figurski, D. H. & Helinski, D. R. Replication of an origin-containing derivative of plasmid RK2 dependent on a plasmid function provided in *trans*. *Proc. Nati. Acad. Sci. USA.* **76,** 1648-1652 (1979).

12. Curson, A. R. J. et al. DSYB catalyses the key step of dimethylsulfoniopropionate

biosynthesis in many phytoplankton. *Nat. Microbiol.* **3,** 430-439 (2018).

13. Kageyama, H., Tanaka, Y., Shibata, A., Waditee-Sirisattha, R. & Takabe, T. Dimethylsulfoniopropionate biosynthesis in a diatom *Thalassiosira pseudonana*: Identification of a gene encoding MTHB-methyltransferase. *Arch. Biochem. Biophys.* **645,** 100-106 (2018).
14. Williams, B. T. et al. Bacteria are important dimethylsulfoniopropionate producers in coastal sediments. *Nat. Microbiol.* **4,** 1815-1825 (2019).
15. Howard, E. C. et al. Bacterial taxa that limit sulfur flux from the Ocean. *Science* **314,** 649-652 (2006).
16. Howard, E. C., Sun, S., Biers, E. J. & Moran, M. A. Abundant and diverse bacteria involved in DMSP degradation in marine surface waters. *Environ. Microbiol.* **10,** 2397-2410 (2008).
17. Howard, E. C., et al. Changes in dimethylsulfoniopropionate demethylase gene assemblages in response to an induced phytoplankton bloom. *Appl. Environ. Microbiol.* **77,** 524-531 (2011).
18. Todd, J. D. et al. Structural and regulatory genes required to make the gas dimethyl sulfide in bacteria. *Science* **315,** 666-669 (2007).
19. Curson, A. R. J., Fowler, E. K., Dicken, S., Johnston, A. W. B. & Todd, J. D. Multiple DMSP lyases in the γ-proteobacterium *Oceanimonas doudoroffii*. *Biogeochemistry.* **110,** 109-119 (2011).
20. Todd, J. D. et al. Molecular dissection of bacterial acrylate catabolism-unexpected links with dimethylsulfoniopropionate catabolism and dimethyl sulfide production. *Environ. Microbiol.* **12,** 327-343 (2010).
21. Curson, A. R., Sullivan, M. J., Todd, J. D. & Johnston, A. W. Identification of genes for dimethyl sulfide production in bacteria in the gut of Atlantic Herring (*Clupea harengus*). *ISME J.* **4,** 144-146 (2010).
22. Curson, A. R. J., Rogers, R., Todd, J. D., Brearley, C. A. & Johnston, A. W. B. Molecular genetic analysis of a dimethylsulfoniopropionate lyase that liberates the climate-changing gas dimethylsulfide in several marine alpha-proteobacteria and *Rhodobacter sphaeroides. Environ. Microbiol.* **10,** 757-767 (2008).
23. Liu, J. et al. Novel insights into bacterial dimethylsulfoniopropionate catabolism in the East China Sea. *Front. Microbiol.* **9,** 3206 (2018).
24. Todd, J. D., Curson, A. R. J., Dupont, C. L., Nicholson, P. & Johnston, A. W. B. The *dddP* gene, encoding a novel enzyme that converts dimethylsulfoniopropionate

into dimethyl sulfide, is widespread in ocean metagenomes and marine bacteria and also occurs in some Ascomycete fungi. *Environ. Microbiol.* **11,** 1376-1385 (2009).

25. Burkhardt, I., Lauterbach, L., Brock, N. L. & Dickschat, J. S. Chemical differentiation of three DMSP lyases from the marine *Roseobacter* group. *Org. Biomol. Chem.* **15,** 4432-4439 (2017).
26. Li, C. Y. et al. Molecular insight into bacterial cleavage of oceanic dimethylsulfoniopropionate into dimethyl sulfide. *Proc. Natl. Acad. Sci. USA.* **111,** 1026-1031 (2014).
27. Todd, J. D., Kirkwood, M., Newton-Payne, S & Johnston A. W. B. DddW, a third DMSP lyase in a model Roseobacter marine bacterium, *Ruegeria pomeroyi* DSS-3. *ISME J.* **6,** 223-226 (2012).
28. Curson, A. R. J., Sullivan, M. J., Todd, J. D. & Johnston, A. W. B. DddY, a periplasmic dimethylsulfoniopropionate lyase found in taxonomically diverse species of Proteobacteria. *ISME J.* **5,** 1191-1200 (2011).
29. Maarel, M., Aukema, W. & Hansen, T. A. Purification and characterization of a dimethylsulfoniopropionate cleaving enzyme from *Desulfovibrio acrylicus*. *FEMS Microbiol. Lett.* **143,** 241-245 (1996).
30. Li, C. Y. et al. Mechanistic insights into dimethylsulfoniopropionate lyase DddY, a new member of the cupin superfamily. *J. Mol. Biol.* **429,** 3850-3862 (2017).
31. Lei, L., Alcolombri, U. & Tawfik, D. S. DddY is a bacterial dimethylsulfoniopropionate lyase representing a new cupin enzyme superfamily with unknown primary function. *bioRxiv* (2017).
32. Curson, A. R. J., Todd, J. D., Sullivan, M. J. & Johnston, A. W. B. Catabolism of dimethylsulphoniopropionate: microorganisms, enzymes and genes. *Nat. Rev. Microbiol.* **9,** 849-859 (2011).
33. Sun, J. et al. The abundant marine bacterium *Pelagibacter* simultaneously catabolizes dimethylsulfoniopropionate to the gases dimethyl sulfide and methanethiol. *Nat. Microbiol.* **1,** 16065 (2016).
34. Li, C. Y. et al. A novel ATP dependent dimethylsulfoniopropionate lyase in bacteria that releases dimethyl sulfide and acryloyl- CoA. *elife* **10,** 1-22 (2021).
35. Alcolombri, U. et al. Identification of the algal dimethyl sulfide–releasing enzyme: A missing link in the marine sulfur cycle. *Science* **348,** 1466-1469 (2015).
36. Carrion, O. et al. A novel pathway producing dimethylsulphide in bacteria is

widespread in soil environments. *Nat. Commun.* **6,** 6579 (2015).

37. Reisch, C. R. et al. Novel pathway for assimilation of dimethylsulphoniopropionate widespread in marine bacteria. *Nature* **473,** 208-211 (2011).

38. Tan, D., Crabb, W. M., Whitman, W. B. & Tong, L. Crystal structure of DmdD, a crotonase superfamily enzyme that catalyzes the hydration and hydrolysis of methylthioacryloyl-CoA. *PLoS. One.* **8,** e63870 (2013).

Source Data for Supplementary Figure 3: Unprocessed SDS-PAGE gels.

Supplementary Figure 3a

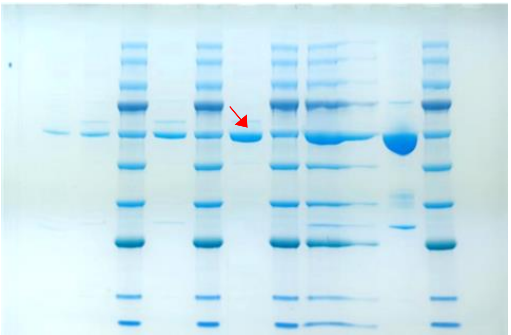

Supplementary Figure 3b

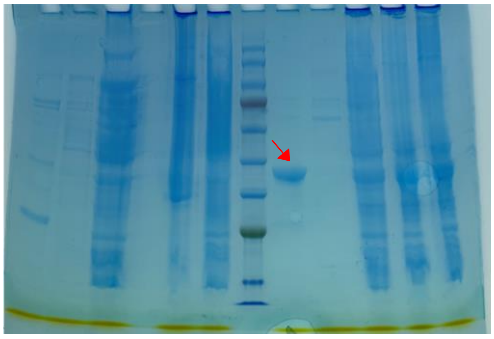

Supplementary Figure 3c

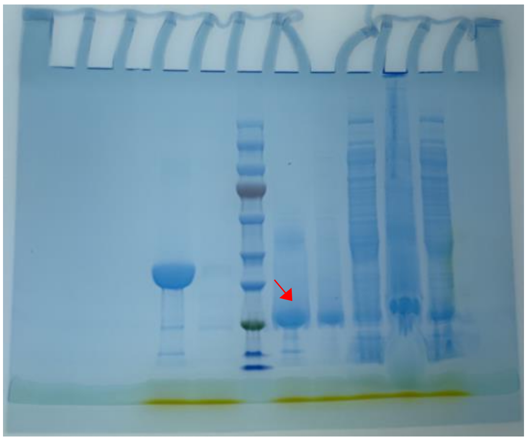

Supplementary Figure 3d

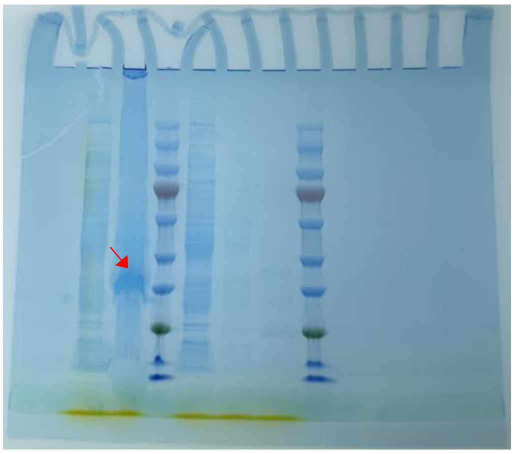

Supplementary Figure 3e

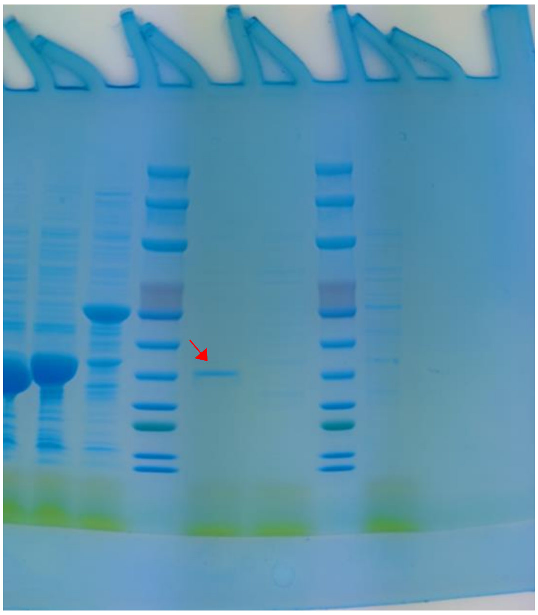

Source Data for Supplementary Figure 9: Unprocessed SDS-PAGE gels.

Supplementary Figure 9a Supplementary Figure 9b

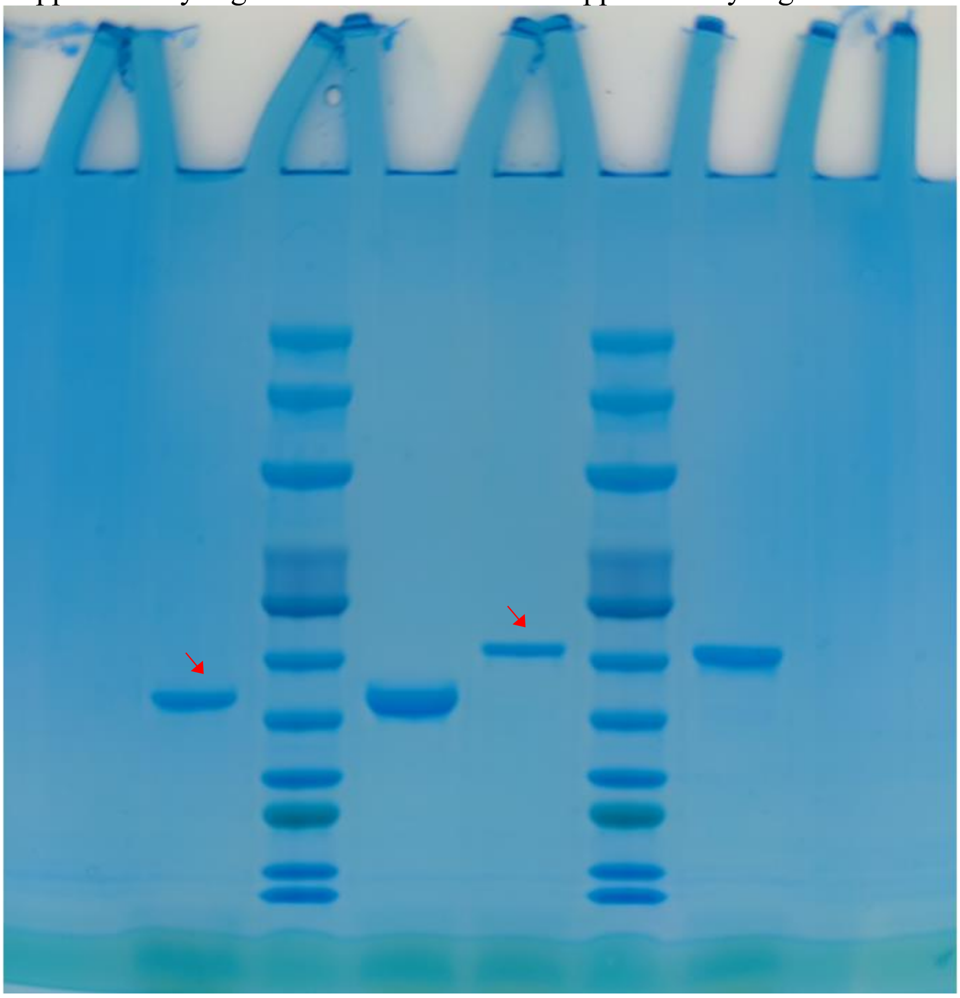

Supplement: Supplementary file 1 — Supplementary Tables 1, 4 and 7–10 and Figs. 1–12. [file 41564_2024_1715_MOESM1_ESM.pdf]
